# Recent Progresses in the Design and Fabrication of Highly Efficient Ni-Based Catalysts With Advanced Catalytic Activity and Enhanced Anti-coke Performance Toward CO_2_ Reforming of Methane

**DOI:** 10.3389/fchem.2020.581923

**Published:** 2020-09-24

**Authors:** Xianyun Wu, Leilei Xu, Mindong Chen, Chufei Lv, Xueying Wen, Yan Cui, Cai-e Wu, Bo Yang, Zhichao Miao, Xun Hu

**Affiliations:** ^1^Jiangsu Key Laboratory of Atmospheric Environment Monitoring and Pollution Control, Collaborative Innovation Center of the Atmospheric Environment and Equipment Technology, School of Environmental Science and Engineering, Nanjing University of Information Science and Technology, Nanjing, China; ^2^College of Light Industry and Food Engineering, Nanjing Forestry University, Nanjing, China; ^3^School of Chemistry and Chemical Engineering, Shandong University of Technology, Zibo, China; ^4^School of Material Science and Engineering, University of Jinan, Jinan, China

**Keywords:** advanced Ni-based catalyst, sintering-proof and anti-coke, carbon dioxide, dry reforming of methane, recent progresses

## Abstract

CO_2_ reforming of methane (CRM) can effectively convert two greenhouse gases (CO_2_ and CH_4_) into syngas (CO + H_2_). This process can achieve the efficient resource utilization of CO_2_ and CH_4_ and reduce greenhouse gases. Therefore, CRM has been considered as a significantly promising route to solve environmental problems caused by greenhouse effect. Ni-based catalysts have been widely investigated in CRM reactions due to their various advantages, such as high catalytic activity, low price, and abundant reserves. However, Ni-based catalysts usually suffer from rapid deactivation because of thermal sintering of metallic Ni active sites and surface coke deposition, which restricted the industrialization of Ni-based catalysts toward the CRM process. In order to address these challenges, scientists all around the world have devoted great efforts to investigating various influencing factors, such as the option of appropriate supports and promoters and the construction of strong metal-support interaction. Therefore, we carefully summarized recent development in the design and preparation of Ni-based catalysts with advanced catalytic activity and enhanced anti-coke performance toward CRM reactions in this review. Specifically, recent progresses of Ni-based catalysts with different supports, additives, preparation methods, and so on, have been summarized in detail. Furthermore, recent development of reaction mechanism studies over Ni-based catalysts was also covered by this review. Finally, it is prospected that the Ni-based catalyst supported by an ordered mesoporous framework and the combined reforming of methane will become the future development trend.

**Graphical Abstract:**
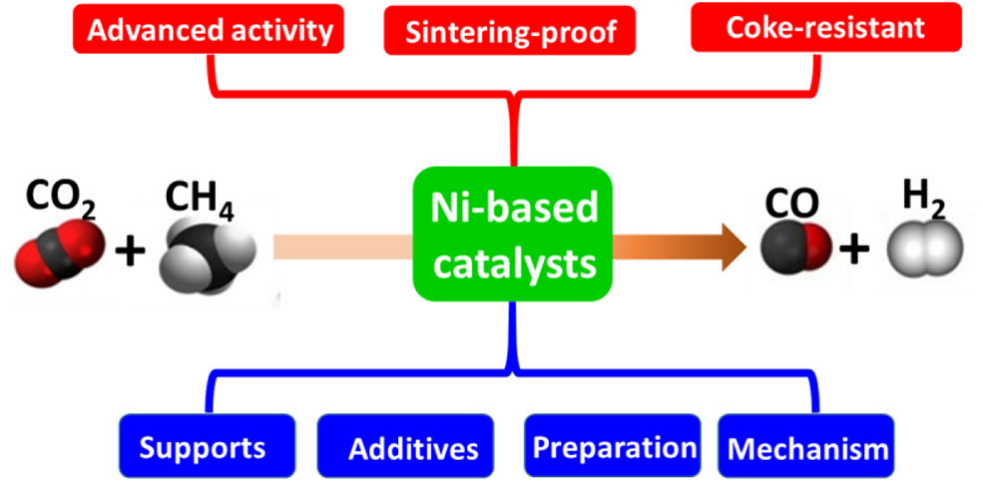
The main outline of this review.

## Introduction

The fossil energy derived from coal, naphtha, and natural gas has become an indispensable part of the development of human society. Therefore, with the rapid development of the human society and economy, fossil energy has been exploited in large quantity (Capellán-Pérez et al., 2014). The consumption of fossil fuels will produce a large amount of CO_2_ and thus cause lots of environmental problems, such as the abnormal climate change and global warming (Zecca and Chiari, 2010). Besides, CH_4_ is the main component of natural gas, which has been extensively considered as the second largest greenhouse gas and has a stronger greenhouse effect than CO_2_ (Jiang et al., 2018). Therefore, research on resource utilization of CO_2_ and CH_4_ is one of the feasible and effective methods to reduce greenhouse gas emissions and global warming (Hernández and Martín, 2019; Ruocco et al., 2019). The CO_2_ reforming of methane can be considered as a promising route to simultaneously utilize these greenhouse gases.

Generally, the reforming of methane can convert methane into syngas, which is the basic unit for the synthesis of high-value chemicals or fuels (Zhao et al., 2014). The reforming of methane can be divided into the following three categories based on the reactants: steam reforming of methane (SRM, CH_4_ + H_2_O → CO + 3H_2_), CO_2_ reforming of methane (CRM, CH_4_ + CO_2_ → 2CO + 2H_2_), and partial oxidation of methane (POM, CH_4_ + 1/2O_2_ → CO + 2H_2_) (Abdullah et al., 2017). Compared with the SRM and POM processes, the CRM process can simultaneously convert CH_4_ and CO_2_ greenhouse gases into the value-added syngas, which would contribute to reducing the greenhouse effect and alleviating the energy crisis (Kim et al., 2019; Dan et al., 2020). The CRM is also an intensively endothermic process owing to the high dissociation energies of CH_4_ and CO_2_ molecules and is carried out under high-temperature conditions according to thermodynamic calculation (de Dios García et al., in press). CRM reaction could produce syngas with a low H_2_/CO molar ratio (H_2_/CO = 1), which is more suitable for further utilization in Fischer–Tropsch synthesis, oxo synthesis, and hydroformylation (Niu et al., 2020). Besides, the CRM has been also considered as the advanced method of storage and transmission of solar or nuclear energy, which drives the endothermic CRM reaction (Delgado Dobladez et al., in press). The product gases are transported to consumers at remote areas, where the reverse exothermic methanation reaction can be carried out and the chemical energy will be released. Compared with SRM and POM, CRM can be considered as the promising process in view of both environmental and economical consideration (Sutthiumporn et al., 2012). However, the CRM process has two major disadvantages and challenges. On the one hand, the methane decomposition reaction (CH_4_ → C + 2H_2_) and Boudouard reaction (2CO → CO_2_ + C) are usually accompanied during the CRM reaction, which will cause the formation of coke deposition over the catalyst surface and thus cause the rapid deactivation of the catalyst (Erdogan et al., 2018). On the other hand, the CRM process is also simultaneously accompanied by the reverse water gas shift reaction (RWGS, CO_2_ + H_2_ → CO + H_2_O) and the coexistence of the RWGS will lead to the actual H_2_/CO ratio lower than the theoretical ratio (1.0) (Barelli et al., 2019; Yentekakis et al., 2019). Besides, the CRM reaction can only be conducted at a high temperature above 640°C based on the thermodynamic calculation due to the high activation energy of CO_2_ and CH_4_ molecules. This will inevitably cause the rapid deactivation of the Ni-based catalyst because of the thermal sintering of the metallic active sites (Chen et al., 2013). As a result, the design and preparation of the Ni-based catalyst with coke-resistant, sintering-proof, and advanced catalytic activity remains a big challenge for the industrial application of the CRM process (Ayodele and Abdullah, 2019).

The CRM catalysts can be mainly divided into two main categories: the noble metal-based catalysts and non-noble metal-based catalysts according to the cost of the active metal components (Lee and Lim, 2020). It is reported that most of the group VIII metals can be used as the active sites of the catalysts toward the CRM process (Horváth et al., 2017). Among these, noble metals, such as Pd, Rh, Pt, and Ru, perform high reactivity, excellent stability, and anti-coking performance in the long-term stability test (Anil et al., 2020). Although noble metal-based catalysts perform excellent catalytic performance, they are not suitable for large-scale industrial application owing to their high cost (Ma et al., 2020). Therefore, worldwide scientists are more interested in seeking non-noble metal-based catalyst alternatives to promote the future industrial application of CRM reaction (Bu et al., 2019; Gao et al., 2020).

Group VIII non-noble metals, such as Fe, Co, and Ni, have been extensively investigated as the effective catalysts for the CRM process (Taherian et al., 2017). Among these metals, the Ni-based catalysts are considered as the most promising catalysts for CRM reaction due to various advantages, such as high catalytic activity and affordable cost (Zhang J. et al., 2020). However, the coke deposition is easily formed over the surface of the Ni-based catalysts at high temperatures, which will make the catalysts to deactivate rapidly because of the coke coverage of the metallic Ni active sites (Xu et al., 2014b). Besides, the metallic Ni nanoparticles easily suffered from thermal sintering due to the low Tammann temperature of the metallic Ni, which also cause the rapid deactivation of the catalysts (Bu et al., 2019). Therefore, the development of Ni-based catalysts with outstanding coke-resistant and sintering-proof performance of the catalysts has gradually become the research focus in the field of CRM.

Generally, basic research and deep understanding of the design and fabrication of Ni-based catalysts will contribute to the configuration of CRM catalysts with excellent activity, sintering-proof property, and coke resistance, which will be beneficial for the successful application in future industry. In recent years, many reviews of CRM reaction catalysts have been reported from different aspects, such as catalyst components (Abdulrasheed et al., 2019), CRM reaction at low temperature (Wang Y. et al., 2018a), and influence of process parameters (Usman et al., 2015). However, the content of the review on this topic is still incomplete, and the prospect of the recent research progresses in the literature review is not enough. Therefore, it is necessary to summarize the recent research progress of nickel-based catalysts with advanced catalytic activity and enhanced anti-coke performance. This review comprehensively summarizes the recent development of Ni-based catalysts with particular attention to the influences of the supports, additives, and preparation methods on the improvement of the catalytic performance of Ni-based catalysts. Besides, the reaction mechanisms of the CRM over Ni-based catalysts are also summarized. Finally, the future development trend of the Ni-based catalysts for the CRM process is prospected.

## Ni-based Catalysts for CRM

The catalytic properties of Ni-based catalysts for CRM reaction are affected by various influencing factors, such as the size of the metallic Ni active site, feature of the support, addition of the promoter addition, and metal–support interaction. Recently, Ni-based catalysts have attracted more and more attention because of their high activities comparable to the noble metals and their low cost (Zhang et al., 2019). However, Ni-based catalysts usually suffer from rapid deactivation due to surface coke deposition and thermal sintering of metallic Ni active sites during the CRM process (Bu et al., 2020). Therefore, great efforts have been devoted to the improvement of surface coke deposition resistance, sintering-proof property of the metallic Ni, and catalytic activity by selecting appropriate support, doping promoted additives, and developing a new preparation method for the catalyst. Several recently developed catalysts for CRM reaction are summarized in Table 1, which differ in terms of the types of supports and promoters, and preparation method.

**Table 1:** Recently developed catalysts for the CRM reaction.

**Catalysts**	**Preparation method**	**GHSV (mL/g h)**	**Temperature (^**°**^C)**	**Time (h)**	**CH_**4**_ conversion (%)**	**CO_**2**_ conversion (%)**	**References**
5Ni/La_2_O_3_-LOC	Impregnation	60,000	700	50	~70	~75	Li et al., 2017
Ni@NiPhy@CeO_2_	Precipitation	36,000	700	85	72.8	79.1	Li and Sibudjing, 2018
Ni/CeO_2_ nanorods	Impregnation	–	700	50	75.4 → 72.8	77.7 → 75.5	Wang N. et al., 2016
Ni/AlSBA-15-EG	Impregnation	18,000	700	20	~75	~83	Zhang et al., 2018
Ni@MA	Impregnation	–	750	4	~76	~88	Arbag, 2018
0.8Co–Ni/CeO_2_	Impregnation	12,000	800	10	77	80	Turap et al., 2020
Ni-MSC-1	Homogeneous precipitation	18,000	800	12.5	85 → 76	93 → 90	Zhang G. et al., 2019
NiO–MgO–Al_2_O_3_	γ-Al_2_O_3_/water interface-assisted method	5,000	800	90	91	89	Chai et al., 2017a
Al_2_O_3_/Ni/Al_2_O_3_ sandwich catalyst	Atomic layer deposition	3,00,000	800	70	92	94	Zhao et al., 2018
20Ni/CeS-1	Impregnation	51,400	750	12	93.57	88.53 → 82.41	Bawah et al., 2018
Ni-La@KCC-1	*In situ* one-pot hydrothermal	–	750	30	~96	~94	Abdulrasheed et al., 2020
M-Ni@SiO_2_	One-pot reverse micelle	–	800	20	~96	~95	Peng et al., 2017
NC-100°C-24 h-C	Reflux-digestion	79,000	850	800	97.5	~91	Chen et al., 2013

### Ni-Based Catalysts With Different Supports

As well-known, catalytic support is considered as an important component of the catalyst by determining the dispersion of the metallic active sites, thereby further influencing the catalytic performance of the catalyst (Sassi et al., 2017). Meanwhile, the metal–support interaction can also greatly affect various features of the catalyst, such as the dispersion, particle size, and metal–support interface, which will further influence the catalytic activity, coke-resistant property, and sintering-proof performances of the catalyst (Bu et al., 2020). Besides, the instinct properties of the support, such as acid–base property and redox property, also influence the activation process of CO_2_, which will further reduce the possibility of the side reactions (Silveira et al., 2017; Azancot et al., 2019). Various materials, such as metal oxides, silica, composite metal oxides, ordered mesoporous materials, zeolites, and carbon materials, have been widely investigated as the supports of Ni-based catalysts toward CRM reaction, which will be presented in the following text.

#### Oxide Supports

##### Single oxide supports

**Figure 1:**
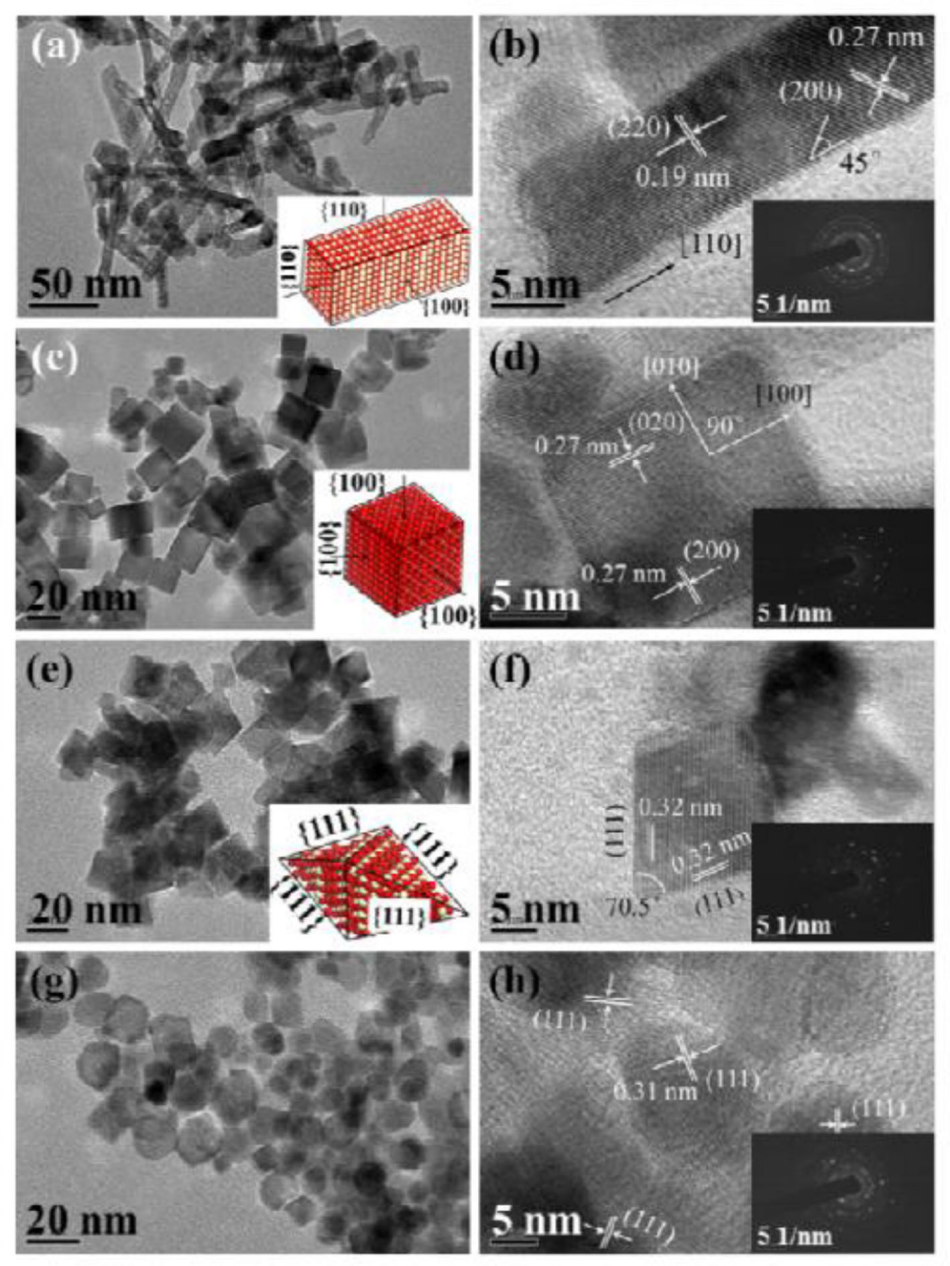
TEM, HRTEM, and SAED (inset) images of as-obtained CeO_2_: **(a,b)** NRs, **(c,d)** NCs, **(e,f)** NOs, and **(g,h)** NPs. Reproduced from Wang N. et al. (2016) with permission from Catalysis Science and Technology.

**Figure 2:**
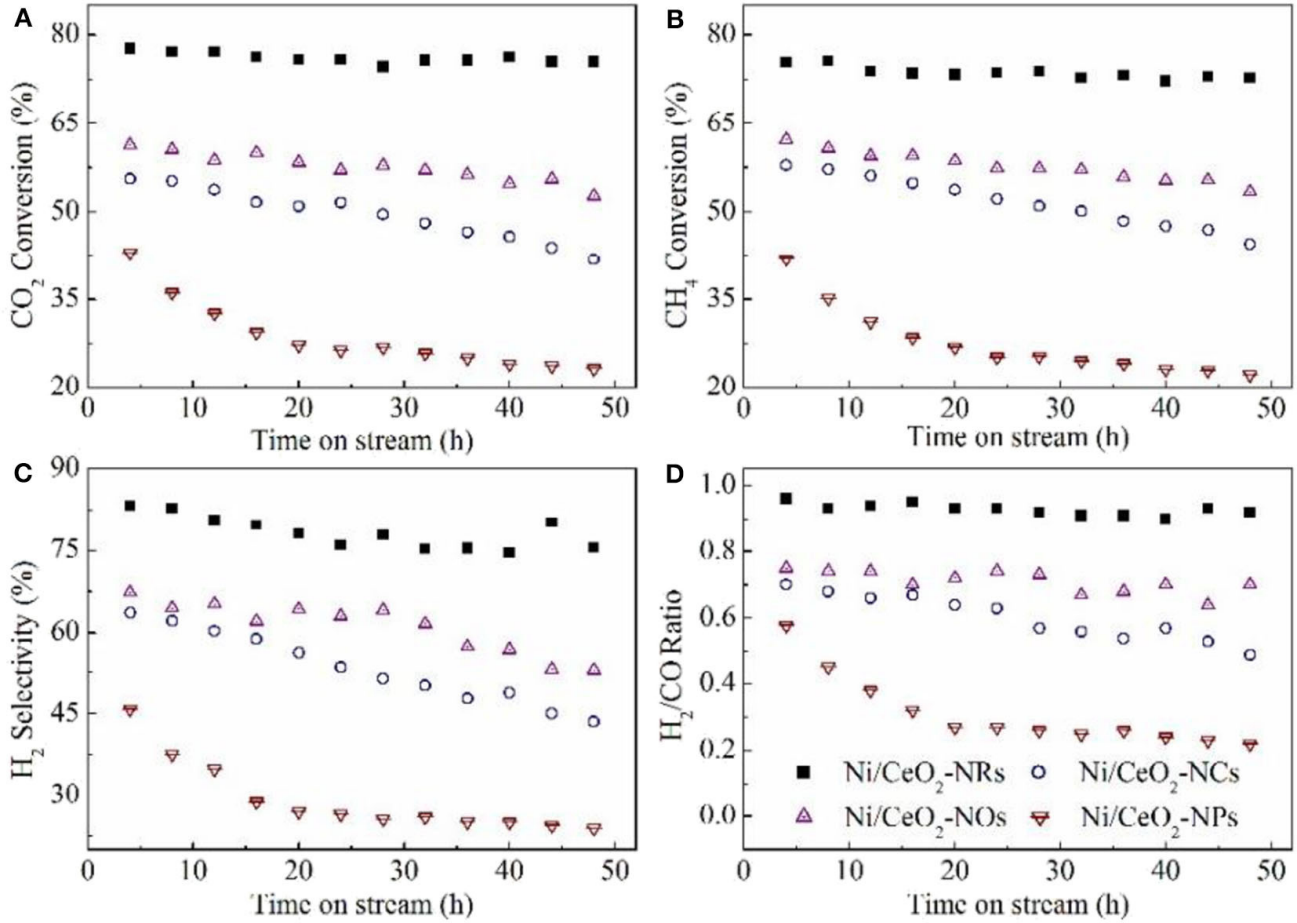
Catalytic stability test of the Ni/CeO_2_ samples for methane dry reforming at 700°C for 50 h. **(A)** CO_2_ conversion; **(B)** CH_4_ conversion; **(C)** H_2_ selectivity; **(D)** H_2_/CO ratio. Reproduced from Wang N. et al. (2016) with permission from Catalysis Science and Technology.

Oxide materials, such as CeO_2_ (Luisetto et al., 2019), SiO_2_ (Zhang L. et al., 2019; de la Cruz-Flores et al., 2020), Al_2_O_3_ (Rahbar Shamskar et al., 2017), MgO (Zhou et al., 2018), and ZrO_2_ (Wang Y. et al., 2018b), are widely utilized as supports of Ni-based catalysts for the CRM process. For example, CeO_2_ has been extensively investigated as a support of Ni-based catalysts because of the formation of a strong metal–support interaction, which can contribute to the enhancement of the reactivity by facilitating the CH_4_ dissociation over the metallic Ni active site (Yan et al., 2019). Besides, the CeO_2_ with a cubic fluorite structure can possess the unique oxidation state through the reversible redox transformation between Ce^4+^ and Ce^3+^ (Luisetto et al., 2019). Specifically, the oxygen formed *via* the CO_2_ dissociation process can be absorbed by oxygen vacancies on the CeO_2_ surface (Tu et al., 2019). Wang N. et al. (2016) synthesized a series of Ni-based catalysts with CeO_2_ supports with four kinds of morphologies (nanorod, nanocube, nanoocta, and nanoparticle, Figure 1) and investigated the influence of the morphology and crystal plane on the catalytic performance of the CRM reaction. As shown in Figure 2, the Ni/CeO_2_-nanorod catalyst displayed the highest catalytic performance and best stability in the CRM reaction among the investigated Ni/CeO_2_ catalysts. Therefore, the structure and catalytic performance of the Ni/CeO_2_ catalyst can be adjusted by tuning the morphology of the CeO_2_ support. Li and van Veen (2018) confirmed that the Ni nanoparticles could be highly dispersed because of the strong metal–support interaction between Ni and CeO_2_. Thus, no serious thermal sintering of the metallic Ni active site occurred during the CRM reaction.

SiO_2_ is widely employed as the support of the Ni-based catalyst owing to its thermally stable feature. Besides, the low surface acidity of SiO_2_ can reduce the coke deposition over the catalyst surface (de la Cruz-Flores et al., 2020). However, it is very difficult to form a strong metal–support interaction over the SiO_2_-supported catalysts, which causes rapid deactivation of the catalysts because of the thermal agglomeration of the metallic Ni active site (Zhao et al., 2016). Therefore, a series of Ni@SiO_2_ core–shell catalysts have been widely investigated in this field (Wang F. et al., 2018). In this system, the SiO_2_ shell can encapsulate the metallic Ni active sites, which can effectively suppress the thermal sintering of the metallic Ni (Wang F. et al., 2016a). Zhang L. et al. (2019) fabricated the Ni@SiO_2_ core–shell catalyst by the microemulsion method with ultrafine Ni nanoparticles (<3.0 nm) for CRM reaction. They found that the catalytic performance of the catalyst was affected by the size of the Ni nanoparticle and the metal–support interaction between Ni and SiO_2_. Besides, the shape of SiO_2_ nanospheres did not obviously change even under high calcination and CRM reaction temperatures, demonstrating good thermal stability. Li et al. (2018) synthesized Ni@Ni phyllosilicate@SiO_2_ core–shell hollow sphere catalysts for CRM reaction by the hydrothermal and reduction method. Ni@NiPhy@SiO_2_ exhibited higher carbon resistance than the Ni@NiPhy catalyst without a SiO_2_ shell at a lower reaction temperature (600°C). The interaction of Ni and NiPhy materials on the Ni@Niphy@SiO_2_ catalyst increased because of the presence of the SiO_2_ shell, which inhibited the growth of Ni nanoparticles. Thus, the growth of CNTs was eliminated and high carbon-resistant performance could be obtained. Das et al. (2018) synthesized an innovative sandwiched core–shell structured Ni-SiO_2_@CeO_2_ catalyst with Ni nanoparticles encapsulated between SiO_2_ and CeO_2_. Ni-SiO_2_@CeO_2_ showed better anti-coking property than Ni-SiO_2_ and Ni-CeO_2_ under low-temperature (600°C) CRM reaction because of the confinement effect of the sandwich structure and the bifunctional mechanism of dry reforming.

As for the Al_2_O_3_ support, it can effectively disperse Ni particles because of its large surface area, favorable thermal stability, and strong metal–support interaction (Rahbar Shamskar et al., 2017). At the same time, the unique acid–base property of Al_2_O_3_ can play a synergistic role with the metallic active site (Shang et al., 2017). Pizzolitto et al. (2019) studied the role of the different kinds of supports in influencing the catalytic performance. They found that the Ni/Al_2_O_3_ catalyst performed much better catalytic stability than did the Ni/CeO_2_ catalyst because of strong metal–support interaction. Xu Y. et al. (2019) compared the catalytic performances of CRM over the Ni/SiO_2_ and Ni/Al_2_O_3_ catalysts. It was found that the Ni/SiO_2_ catalyst suffered rapid deactivation because of the weak metal–support interaction, which caused the thermal sintering of the metallic Ni and further accelerated the formation of the coke. As a comparison, the Ni/Al_2_O_3_ catalyst performed much better catalytic stability because of the strong metal–support interaction. This would facilitate the formation and stabilization of small Ni nanoparticles and further reduce the surface coke deposition over the Ni/Al_2_O_3_ catalyst.

Similar to Al_2_O_3_, ZrO_2_ also possesses both basic and acidic properties and is widely used as the catalytic support (Shin et al., 2018). As a support, it can form the strong metal–support interaction with the Ni active site. Therefore, the metallic Ni nanoparticles can be well-dispersed on the support, which is further conducive to the improvement of the catalytic activity and anti-coke property of the catalyst (Nabgan et al., 2020). Wang Y. et al. (2018b) confirmed that the metal–support interaction of the Ni-Si/ZrO_2_ catalyst not only facilitated the formation of small metallic Ni active sites with strong electron donor capability but also maintained the state of the Ni particles under CRM reaction conditions.

MgO is widely employed as the support of the Ni-based catalyst owing to its thermally stable feature and low cost (Zanganeh et al., 2013). Besides, its strong Lewis basicity catalyst has a strong chemisorption effect toward CO_2_ and can inhibit coke deposition by accelerating the coke-eliminating reaction (Zhang et al., 2018). Zuo et al. (2018) used theoretical modeling and experimentation to study the catalytic performance of single-site Ni/MgO catalysts in CRM reactions. As the particle size of Ni increases, the single-site Ni/MgO catalyst provided stronger bindings and more sufficient sites to activate CH_4_ and CO_2_. Song et al. (2020) evaluated the catalytic activity of a Ni–Mo nanocatalyst, which were stabilized at the edges of a single-crystalline MgO. NiMoCat showed stable activity and high conversion rates of CH_4_ and CO_2_ in 850 h of continuous running test. The activity values of NiMoCat were much greater than many traditional catalysts.

**Figure 3:**
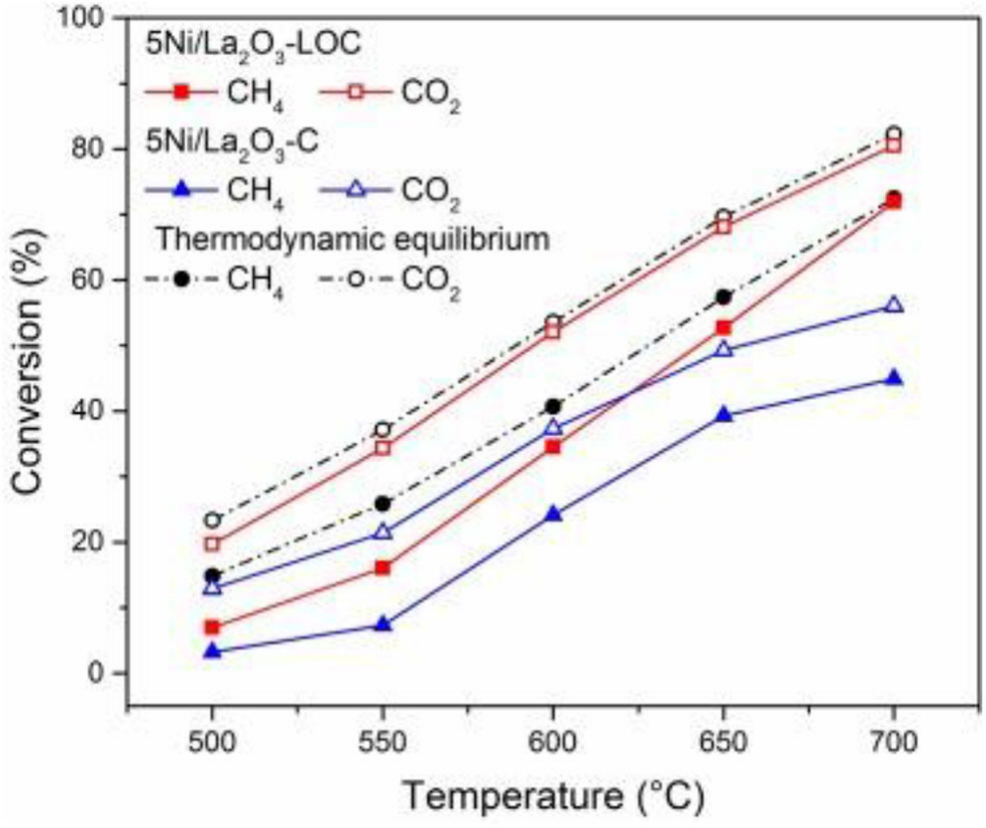
DRM activity of the catalysts. Reaction conditions: CH_4_/CO_2_/N_2_ = 15/15/70 mL/min, GHSV = 60,000 mL h^−1^ ${\text{g}}_{\text{cat}}^{-1}$, 1 atm. Reproduced from Li et al. (2017) with permission from Applied Catalysis B: Environmental.

As an alkaline support, La_2_O_3_ is also conducive to the adsorption and activation of CO_2_ on the surface of Ni-based catalysts (Li et al., 2017). Meanwhile, it can react with CO_2_ to produce La_2_O_2_CO_3_, thereby reacting with coke on the surface of nearby Ni nanoparticles to produce CO and regenerate La_2_O_3_ (Tsoukalou et al., 2016; Xu L. et al., 2019). Xu L. et al. (2019) used various methods, such as glycine nitrate combustion (GNC), precipitation (PP), and thermal decomposition (TD) to prepare La_2_O_3_ supports with different bulk and surface properties to study the structure–reactivity relationship of Ni/La_2_O_3_. Among these catalysts, the 5Ni/La_2_O_3_-GNC catalyst with the most surface alkaline and active oxygen sites displayed the best catalytic activity and stability. Li et al. (2017) tested the catalytic activity of the Ni/La_2_O_3_-LOC catalyst synthesized with La_2_O_2_CO_3_ nanorod as the support precursor. As shown in Figure 3, the activity of 5Ni/La_2_O_3_-LOC is higher than that of 5Ni/La_2_O_3_ because the nanorod support of 5Ni/La_2_O_3_-LOC improved the dispersion of nickel particles and increased the number of exposed active sites. They also found that the dispersion of Ni affected the distribution of coke and La_2_O_2_CO_3_ on the catalyst surface, which in turn affected the catalytic performance of the catalyst. Gao et al. (2018) doped the La_2_O_3_ into carbon fibers (CF) as a support to prepare the NI/CF-LA_2_O_3_ catalyst. The addition of La_2_O_3_ improved the activity of the Ni/CF catalyst because La_2_O_3_ could improve the dispersion of Ni and enhance the metal–support interaction between Ni and support. In addition, the conversion of La_2_O_3_ to La_2_O_2_CO_3_ affected the removal rate of deposited carbon and improved the catalyst activity of Ni/CF-La_2_O_3_.

##### Composite oxides

**Figure 4:**
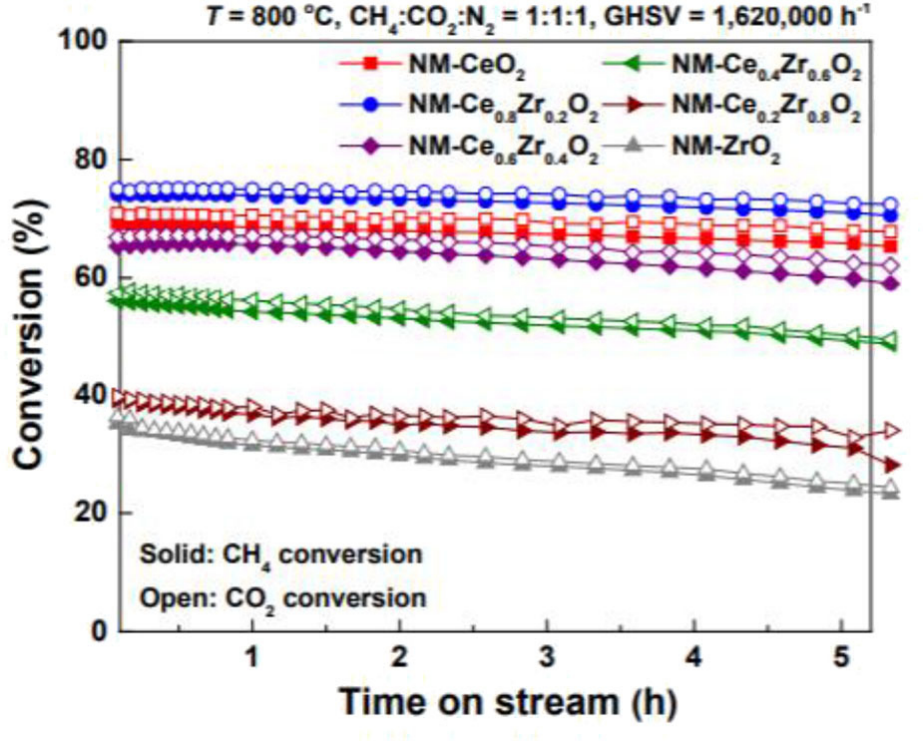
CH_4_ and CO_2_ conversions with time on stream (TOS) over NMCeO_2_, NM-ZrO_2_, and NM-Ce_(1−x)_Zr_(x)_O_2_ catalysts in CRM reaction (T = 800°C, CH_4_:CO_2_:N_2_ = 1:1:1, and GHSV = 1,620,000 mL·g^−1^·h^−1^). Reproduced from Jang et al. (2018) with permission from Green Chemistry.

In addition to the single oxides, the composite oxides are also often used as the supports of the Ni-based catalysts for CRM reactions because it can overcome the disadvantages of the single oxides and further improve the catalytic performance of the catalysts (Shah et al., 2019). For example, the CeO_2_ support possesses excellent redox properties; however, its thermal stability was relatively poor, especially under CRM conditions (Long et al., 2020). In order to address this challenge, doping another metal cation into the crystal structure of CeO_2_ or depositing another basic metal oxide (such as La_2_O_3_, Al_2_O_3_, ZrO_2_) over the CeO_2_ surface has been considered as an effective strategy (Padi et al., 2020). ZrO_2_ has excellent thermal stability at high temperature and thus the introduction of Zr^4+^ cation into the CeO_2_ lattice to form a CeO_2_-ZrO_2_ solid solution can greatly improve the oxygen storage capacity, oxygen mobility, and thermal stability, which further enhances the anti-coke property (Shi et al., 2019). The partial substitution of Ce^4+^ by Zr^4+^ causes the shrinkage and deformation of the CeO_2_ lattice and then more oxygen vacancies are formed, which induces enhanced mechanical properties and ion conductivity (Wang Y. et al., 2019). Furthermore, the low concentration of Lewis acid sites on the surface of the CeO_2_-ZrO_2_ solid solution support can effectively reduce the coke deposition caused by the surface acidity (Xu et al., 2012a). Dudek et al. (2019) evaluated the effect of CeO_2_-ZrO_2_ support prepared by supercritical fluid method on the performance of Ni-based catalysts. The NiO_x_/CeO_2_-ZrO_2_ catalyst displayed favorable thermal stability and catalytic performance. They also found that the employment of the continuous supercritical preparation techniques to prepare Ni-based catalysts could improve its resistance to deactivation. Jang et al. (2018) compared the catalytic performance of Ni-based catalysts supported on CeO_2_, ZrO_2_, and CeO_2_-ZrO_2_ solid solution toward the CRM reaction. As shown in Figure 4, the Ni-MgO-Ce_0.8_Zr_0.2_O_2_ catalyst performed the best stability and catalytic activity due to the highest degree of reduction and the smallest metallic Ni particle size. CeO_2_-Al_2_O_3_ composite oxide is also considered as an excellent support for CRM reaction because the presence of CeO_2_ can greatly improve the metal–support interaction and the stability of Al_2_O_3_ at high temperature (Stroud et al., 2018). According to the previous report, the CeO_2_-Al_2_O_3_ support is beneficial to dispersing the active sites, improving the reducing ability of the catalyst, and enhancing the oxygen flow (Jawad et al., 2019).

For TiO_2_-Al_2_O_3_, TiO_2_ can effectively prevent coke deposition because of its reducible properties (Kim et al., 2015). At the same time, the presence of Al_2_O_3_ with a high surface area can overcome the shortcomings of TiO_2_ support, such as the low surface area and the change of crystalline phase during the CRM reaction (Shah et al., 2020). Shah et al. (2019) compared the catalytic activity and stability of Ni-based catalysts with different TiO_2_/Al_2_O_3_ molar ratios. The results of their study indicated that the synergistic effect of TiO_2_ and Al_2_O_3_ prevented the formation of coke and the occurrence of side reactions. The catalyst with the most suitable doping ratio of TiO_2_ and Al_2_O_3_ could improve the catalytic activity and stability of the catalyst. Shah et al. (2020) changed the synthesis parameters to design and optimize the nanocrystalline mesoporous Ni-based catalyst toward CRM reaction. They found that the TiO_2_-Al_2_O_3_ mixed oxide was the main driving force for the sustainable activity of the catalyst.

For MgO-Al_2_O_3_, it was found that the interaction between the active metal and the support was enhanced by the formation of MgAl_2_O_4_, thus improving the dispersion of Ni on the support and reducing the catalyst deactivation caused by coking (Bach et al., 2020). Jin et al. (2017) prepared a carbon-Ni/MgO-Al_2_O_3_ composite catalyst by using sucrose as the precursor and tested its catalytic performance toward CRM reaction. The results showed that the composite catalyst performed good coking resistance and could inhibit the growth of metallic Ni active sites. They also found that the carbonization temperature of the composite catalyst precursor can affect the activity of this catalyst. Chai et al. (2017a) synthesized a sort of NiO-MgO-Al_2_O_3_/FeCrAl-fiber catalyst with a microfiber structure for DRM reaction, and the obtained catalyst showed excellent catalytic activity and stability. The Ni nanoparticles in the catalyst were evenly nested in the MgO-Al_2_O_3_ nanosheet composites, which was crucial for the formation of rapid carbon filaments.

#### Zeolite Supports

**Figure 5:**
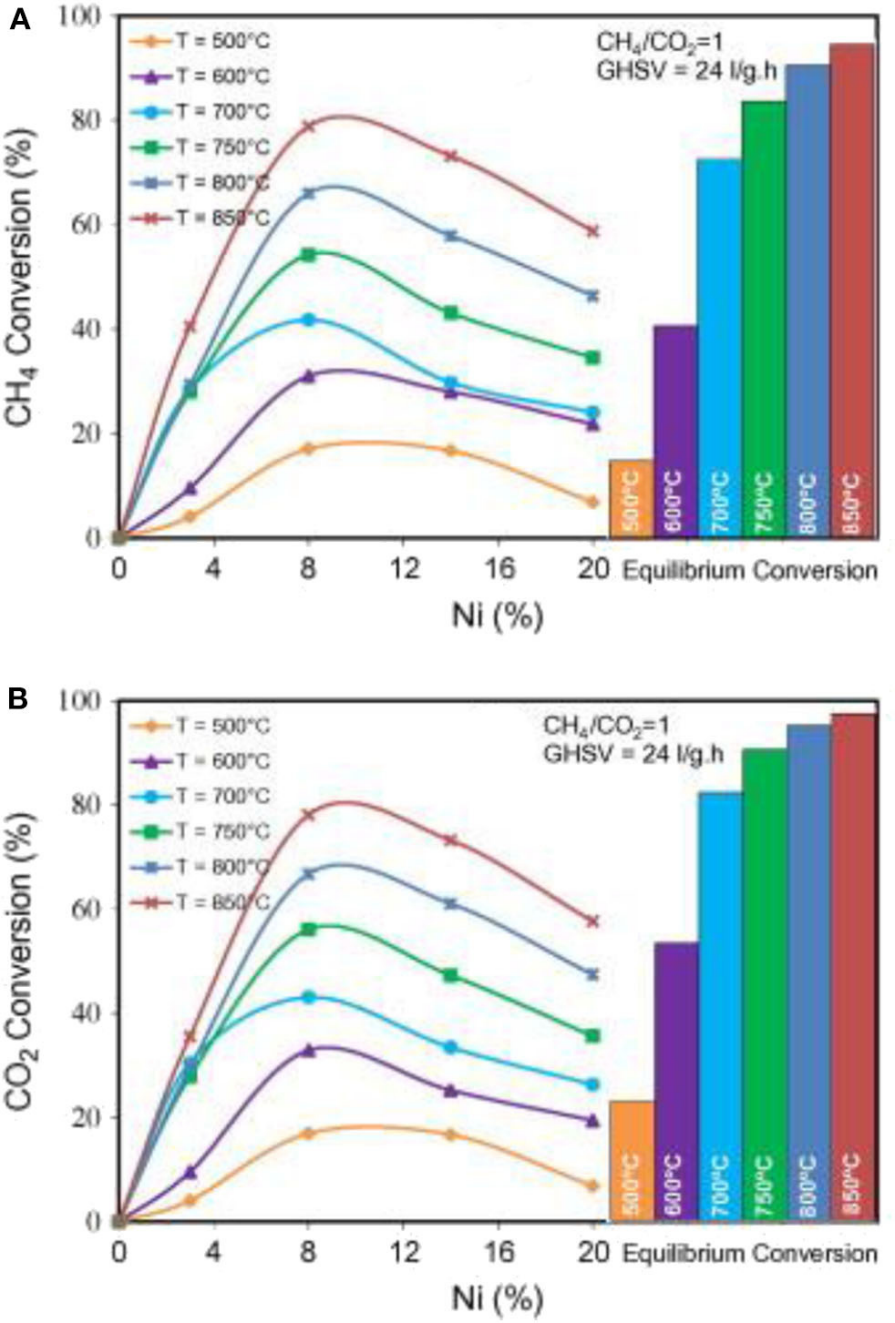
Effect of Ni loading on CH_4_ **(A)** and CO_2_ **(B)** conversion at different temperatures over nanostructured Ni/ZSM-5 catalysts synthesized via the ultrasound method. Reproduced from Vafaeian et al. (2013) with permission from Energy Conversion and Management.

Zeolites are a group of crystalline aluminosilicate materials with microporous structures and are widely investigated as supports of the Ni-based catalysts of the CRM reaction because of their advantages of abundant microporous channels, good thermal stabilities, large specific surface areas, and big pore volumes (Martínez Galeano et al., 2019; Kumar et al., 2020; Zhu et al., 2020). The Si/Al ratio of zeolite reflects the number of charge compensating ions and can modify the surface acidity (Li W. et al., 2019). In view of these advantages, the zeolites have been widely investigated as catalytic supports of metal or oxide clusters in many catalytic processes owing to the high metal dispersions and excellent stabilities (Xie et al., 2019). Moradi et al. (2016) confirmed that the Si/Al ratio plays an important role in affecting the catalytic performance of the nano-Ni/ZSM-5 catalyst in the CRM reaction. They prepared a series of Ni/ZSM-5 nano-catalysts with different Si/Al ratios. The experimental results showed that the acid catalyst with the lowest Si/Al ratio [5NZ(30)] had excellent catalytic performance and coke resistance in the CRM process. They also found that the specific surface area and pore volume of ZSM-5 zeolite would increase as the Si/Al ratio decreased. Vafaeian et al. (2013) prepared a series of Ni/ZSM-5 nanocatalysts with different Ni loading amounts by the ultrasonic assisted method. As shown in Figure 5, the Ni/ZSM-5 nanocatalyst with the Ni content of 8% showed excellent catalytic activity because of its high specific surface area, uniform particle size distribution, and high dispersion of metallic Ni active sites. Estephane et al. (2015) investigated the catalytic performances of ZSM-5-supported Ni and/or Co monometallic and bimetallic catalysts prepared by the wet impregnation method. Among these catalysts, the bimetallic catalyst with high Co loading amounts (1Ni2Co/ZSM5) performed the best catalytic activity and the lowest coke deposition after 12 h of operation.

**Figure 6:**
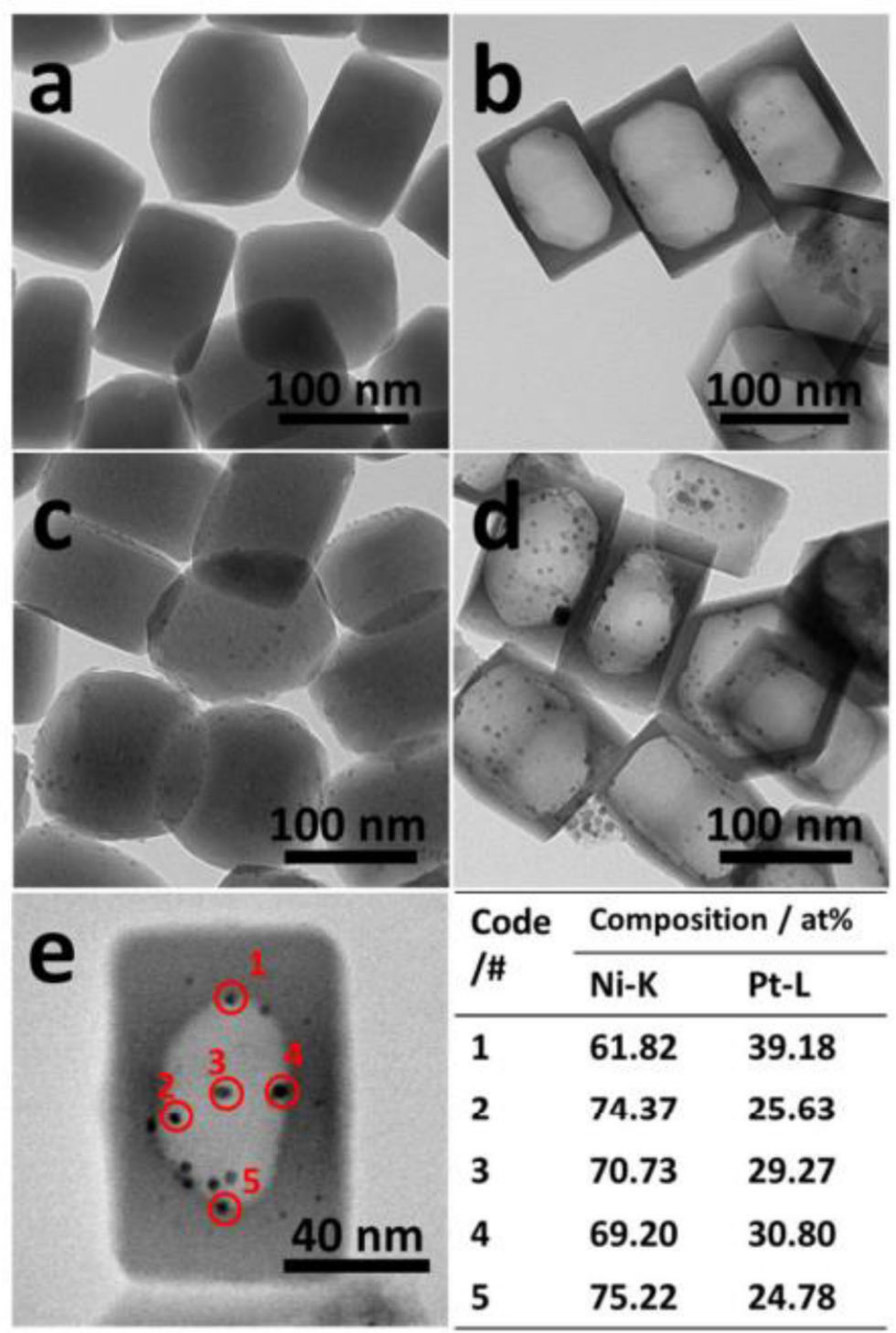
TEM images of prepared **(a,c)** 1.5NiO-0.5Pt/S-1 and **(b,d)** 1.5NiO-0.5Pt@Hol S-1 after calcination in air at 400°C for 2 h **(a,b)** and reduction under H_2_ at 800°C for 30 min **(c,d)**. **(e)** STEM image of prepared 1.5NiO-0.5Pt@Hol S-1 after reduction under H_2_ at 800°C for 30 min, and quantified phase compositions in the Ni-Pd nanoparticles from STEM maps. Reproduced from Dai et al. (2015b) with permission from *Journal of Materials Chemistry A*.

Silicalite-1 (S-1) is a sort of zeolite with weak acidity owing to no alumina component in the zeolite framework (Wei et al., 2017). The diffusion rates of H_2_, CO, CO_2_, and CH_4_ in S-1-supported catalyst are very fast (Laprune et al., 2017). Bawah et al. (2018) confirmed that mesoporous Na-silicalite-1 (MFI) zeolite was a suitable support material for the CRM reaction catalyst. The ion exchange between MFI and Ce significantly increased the mesopore volume, improved the activity of Ni/silicalite-1, and reduced the formation of carbon deposition. The reduction in diffusion restrictions and the high surface area made the sintered Ni particles smaller and the catalytic activity of the catalyst increased. In recent years, hollow zeolite has been widely used in CRM reactions due to its excellent properties in product selectivity, anti-active site leaching, and anti-metal sintering (Dai et al., 2015a). Dai et al. (2015b) confirmed that the 1.5Ni-0.5 Pt@Hol S-1 catalyst obtained by encapsulating highly dispersed Ni-Pt bimetallic nanoparticles in hollow silicalite-1 single crystals performed good anti-coking and sintering properties in CRM reactions. As shown in Figure 6, Ni was wrapped in hollow zeolite crystals and the small Ni particles inhibited the formation of coke. The encapsulation shell prevented coke formed on the outside from reducing the activity of the Ni inside, resulting in a stable and high activity even in the presence of carbon.

#### Carbon Supports

Carbon materials, such as activated carbon (AC) (Wang H. et al., 2019), carbon nanotubes (CNT) (Figueira et al., 2018), and carbon fiber (CNF) (Chai et al., 2017b), have been widely used as supports of the Ni-based catalysts of the CRM reaction. Because the carbon-based materials usually have lots of advantages, such as large surface areas, excellent stability, chemical inertness, and low cost (Li et al., 2020). Their pore-size distributions and surface functionalities could be readily tuned by controlling specific parameters (Sun et al., 2018). In addition, the recovery of active metals from spent catalysts can be achieved and recycled by burning the carbon supports, especially for the precious metal-based catalysts (Khavarian et al., 2014). Besides, the surface oxygen-containing functional groups can be formed after the oxidation treatment of AC. The surface oxygen-containing functional groups can act as nucleation centers, which is beneficial to the high dispersion of the metallic active site over the surface of AC (Forouzesh et al., 2019). Tan et al. (2019) compared the difference between the wood-derived activated carbon (WAC) and coal-derived activated carbon (CAC) as the catalysts toward the microwave-assisted CRM. The results demonstrated that WAC performed much better catalytic activity toward the CRM reaction. The reason for this was that the WAC had a larger specific surface area and bigger specific pore volume than the CAC. Thus, WAC could provide more accessible active sites, which was conducive to the adsorption and diffusion of gas. Wang H. et al. (2019) reported that the synergistic effect between the Ni/AC and plasma could greatly improve the catalytic activity toward CRM reaction by intensifying the Ni-AC metal–support interaction. Sun et al. (2019b) prepared Co–M/AC–N (M = Ce, Fe, Zr) catalysts by co-impregnation and evaluated their catalytic performances toward CRM reaction. They found that the doping of N resulted in a higher Co^2+^/Co^3+^ ratio and improved the stronger interaction between Co and Ce. Therefore, the addition of N could increase the catalytic activity of the catalyst.

**Figure 7:**
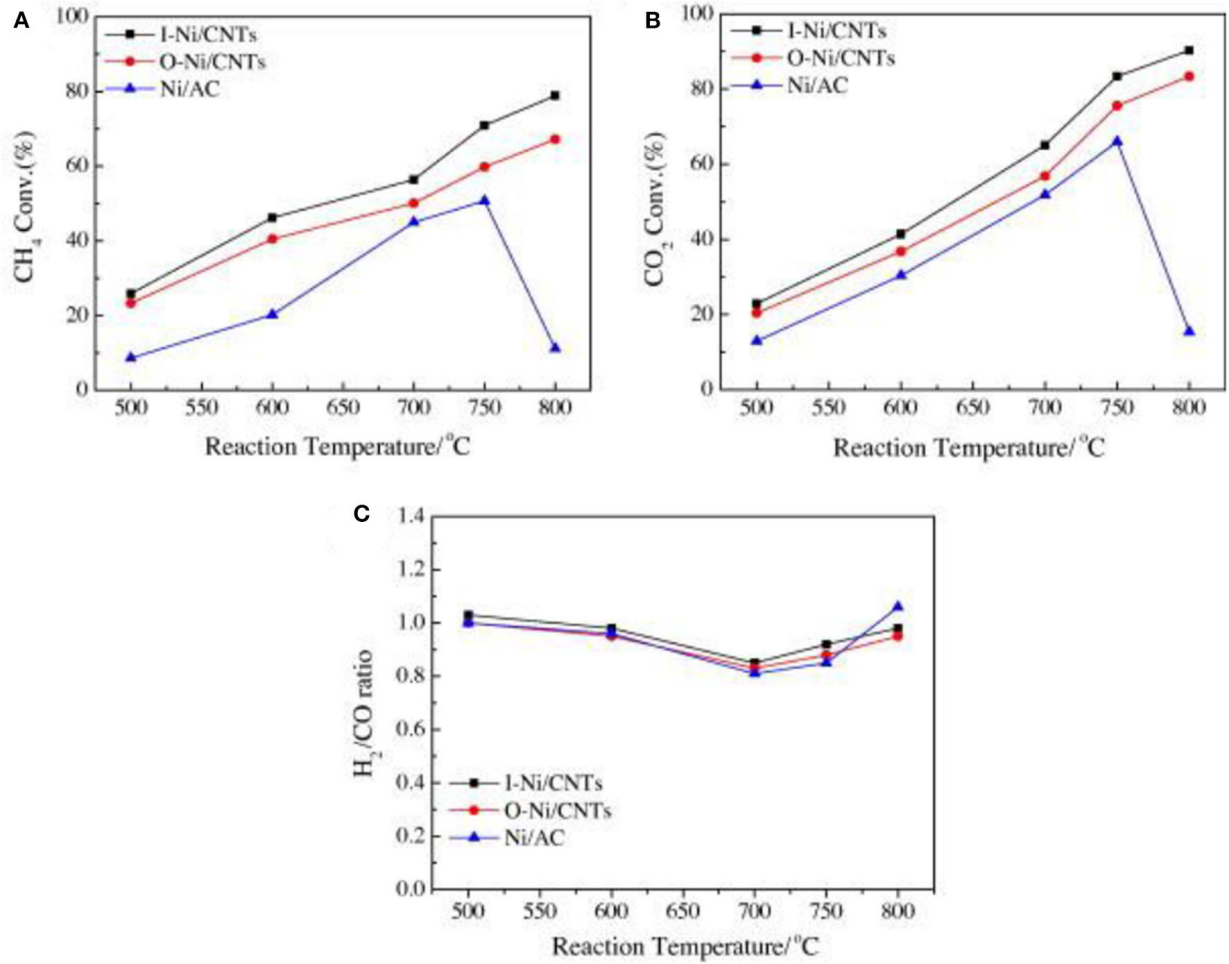
The conversion–temperature relationships of Ni/AC, O–Ni/CNT, and I–Ni/CNT catalysts. **(A)** CH_4_ conversion; **(B)** CO_2_ conversion; **(C)** H_2_/CO ratio (reaction conditions: W/F = 1 g h/mol). Reproduced from Ma et al. (2013) with permission from fuel.

Carbon nanotube (CNT), well-known as a new nano-carbon catalyst support, possesses many advantages, such as large surface area, unique electronic properties, tubular structure, chemical inertness, thermal stability, and high mechanical strength (Figueira et al., 2018). Therefore, it can effectively improve the dispersion of metal particles and enhance the adsorption of reactants (Figueredo et al., 2018). Ma et al. (2013) prepared a series of catalysts for the CRM reaction of Ni nanoparticles loaded inside or outside CNTs and studied the effect of catalytic sites on the surface of CNTs. As shown in Figure 7, the metallic Ni nanoparticles loaded inside CNT catalysts showed the best catalytic activity among these catalysts. This could be attributed to the difference in electronic density between interior and exterior surfaces of CNTs and the confinement effect of CNTs. Donphai et al. (2014) prepared the composites between Ni and CNTs over mesocellular silica support (Ni-CNTs/MS) for CRM reactions. The results showed that the Ni-CNTs/MS catalyst had better stability than the Ni/MS catalyst due to selective formation of carbon by-products as the tube-length extension of the existing CNTs.

As for carbon fiber, it has a similar thermal stability and high specific surface area to the activated carbon. Besides, their fibrous properties make them more beneficial to producing structurally defined catalysts (Wei et al., 2020). Wei et al. (2020) demonstrated that the carbon fiber with layered silicate could improve the sintering performance of the Ni-based catalyst at high temperature of CRM because carbon fiber favored the decomposition of CO_2_. Yu et al. (2018) prepared Ni-based catalysts with different ratios of Al_2_O_3_/ACF for CRM reaction. They found that the incorporation of the activated carbon fibers (ACF) to the Ni/γ-Al_2_O_3_/ACF catalyst was beneficial to improvement of the CRM catalytic activity. ACF could reduce the grain size of NiAl_2_O_4_ spinel in Ni-based catalysts, which was conducive to the conversion of methane. The addition of ACF could provide abundant functional groups on the catalyst surface and effectively promote the catalytic performance of the CRM reaction.

#### Ordered Mesoporous Materials

Ordered mesoporous materials are extensively employed as supports of the Ni-based catalysts for CRM reaction because of their outstanding advantages in textural properties, such as large specific surface areas, tunable pore sizes, and big volumes, which are beneficial to the high dispersion of the active sites (Xu et al., 2013). Furthermore, the confinement effect of the mesopore can effectively suppress the growth of metallic Ni nanoparticles within the size of the mesopore (Xu et al., 2016b). As a result, the metallic Ni active sites with uniform size distribution can be formed and the thermal sintering of the metallic Ni active sites can be inhibited during the CRM reactions (Xu et al., 2012a; Al-Fatesh et al., 2018). Among them, the ordered mesoporous metal (composite) oxides and ordered mesoporous silica material have been widely investigated as catalytic supports in CRM reaction (Xu et al., 2016b; Bukhari et al., 2017).

**Figure 8:**
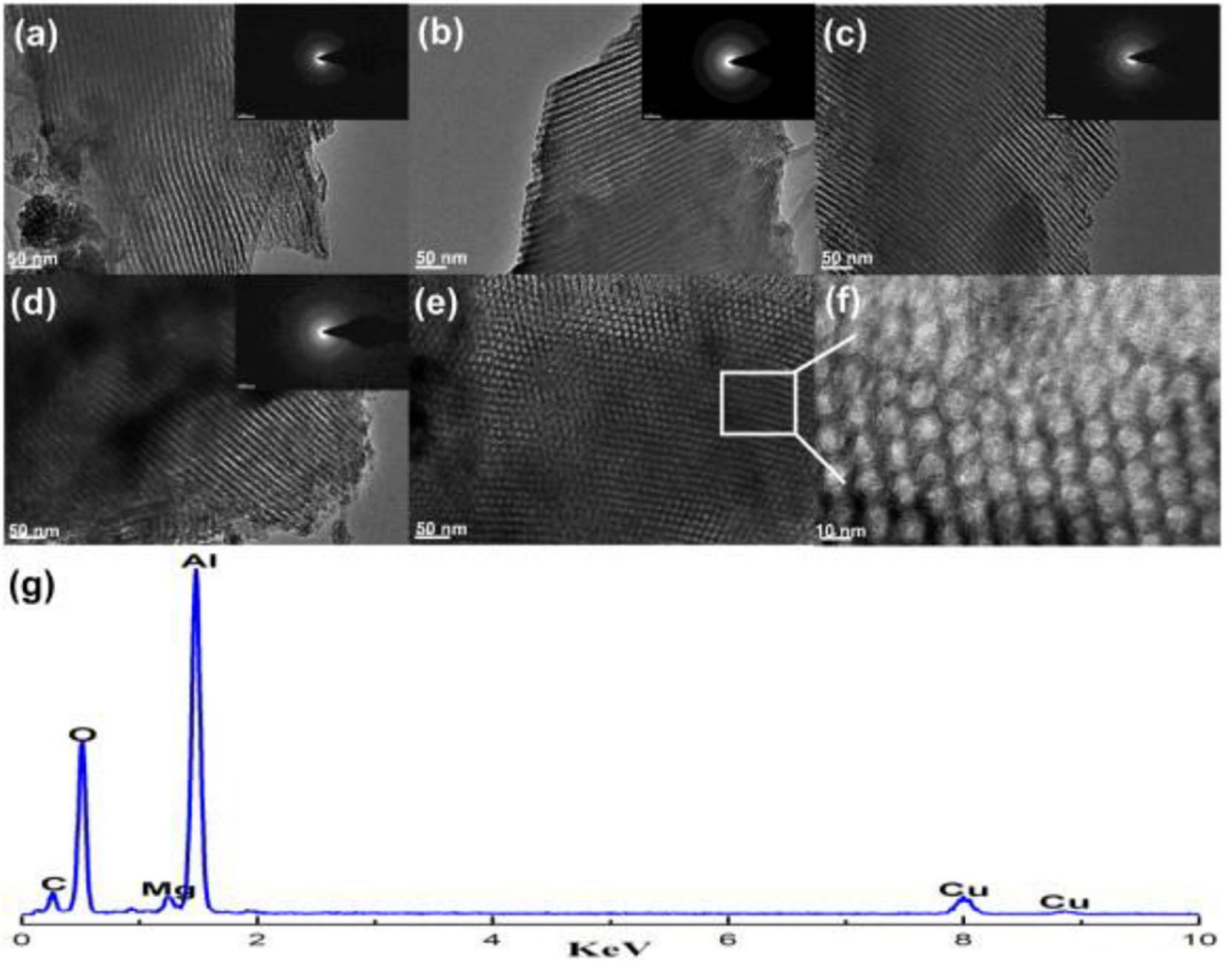
TEM and SAED images of the OM-*x*Mg*y*Al materials calcined at 700°C: **(a)** OM-3Mg97Al, **(b)** OM-5Mg95Al, **(c)** OM-8Mg92Al, **(d–f)** OM-10Mg90Al, and **(g)** EDX measurement for OM-5Mg95Al. Reproduced from Xu et al. (2013) with permission from International *Journal of Hydrogen Energy*.

**Figure 9:**
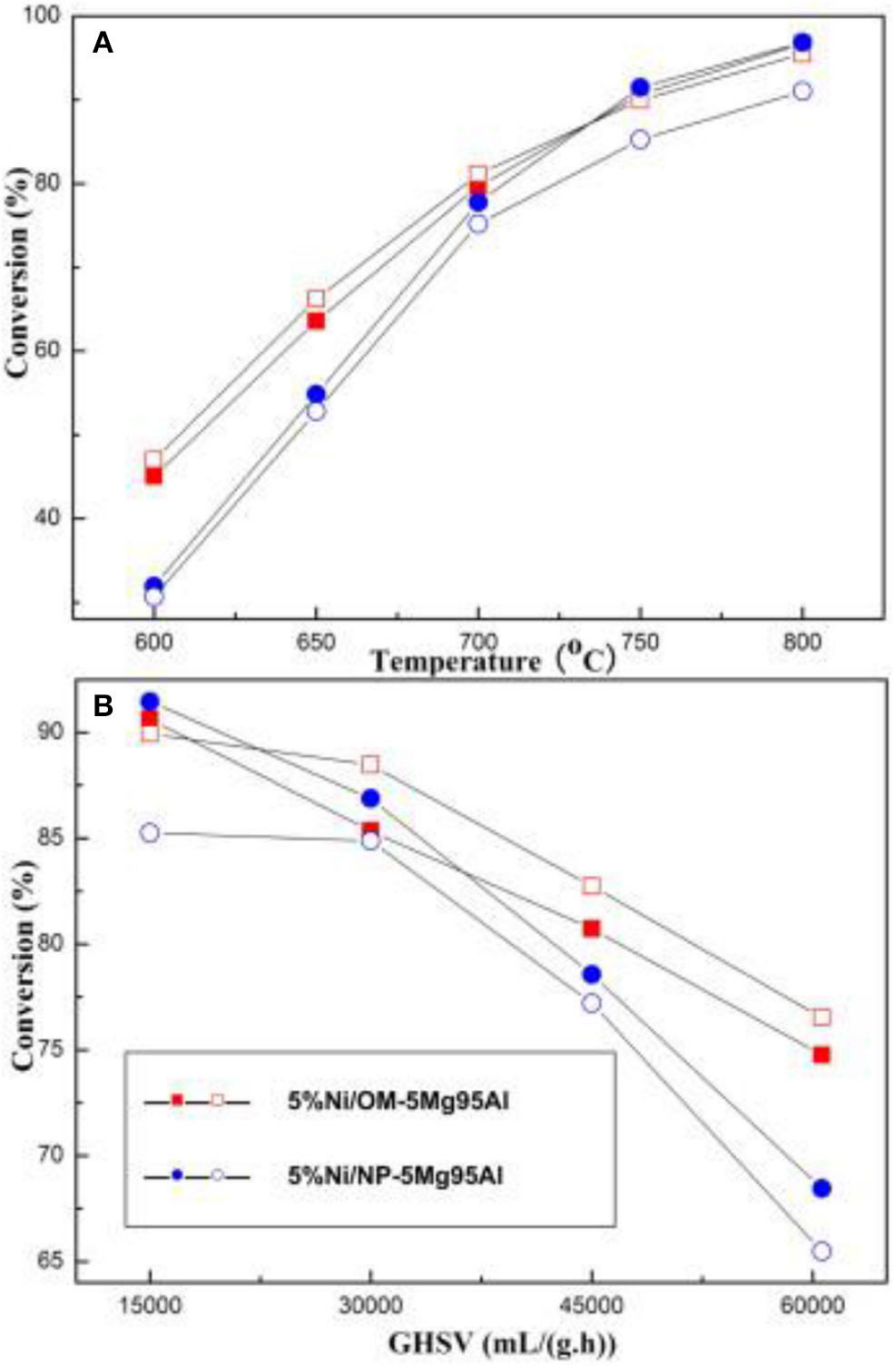
CH_4_ and CO_2_ conversions over the 5%Ni/OM-5Mg95Al and 5%Ni/NP-5Mg95Al catalysts under various reaction conditions: **(A)** T = 600–800°C, CH_4_/CO_2_ = 1, GHSV = 15,000 mL/(g h), 1 atm; **(B)** GHSV = 15,000–60,600 mL/(g·h), CH_4_/CO_2_ = 1, 750°C, 1 atm. Reproduced from Xu et al. (2013) with permission from *International Journal of Hydrogen Energy*.

**Figure 10:**
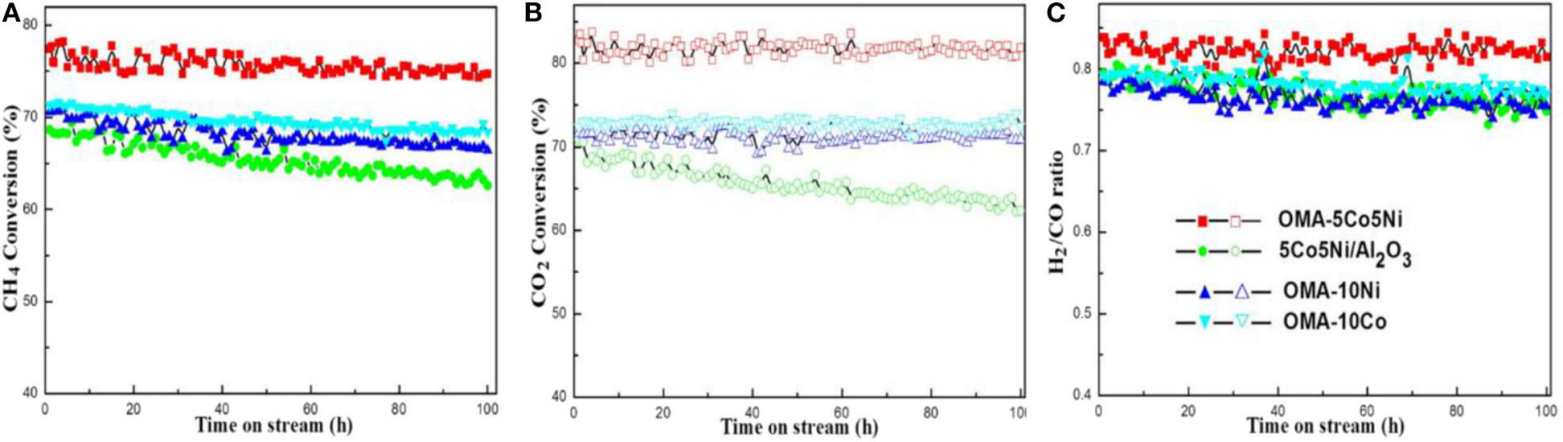
Long-term stability tests (100 h) over different catalysts: **(A)** CH_4_ conversion, **(B)** CO_2_ conversion, **(C)** H_2_/CO ratio; reaction conditions: CH_4_/CO_2_ = 1, GHSV = 15,000 mL/(g h), 700°C, 1 atm. Reproduced from Xu et al. (2016a) with permission from *International Journal of Hydrogen Energy*.

Recently, the ordered mesoporous alumina with high thermal stability and outstanding structural property have attracted more and more attention (Xu et al., 2016a). Li B. et al. (2019) incorporated the secondary metal (M = Fe, Co, Cu) into the Ni-based ordered mesoporous alumina catalyst (MNiAl) for CRM reaction. Among these catalysts, FeNiAl displayed the best catalytic activity owing to the formation of FeNi_3_ alloy during the CRM reaction. Xu et al. (2012c) prepared a series of Ni-based catalysts supported on ordered mesoporous alumina (OMA) for CRM reaction. These catalysts exhibited excellent catalytic activity and stability. They confirmed for the first time that the mesoporous frameworks of these catalysts could provide more “accessible” Ni active centers and effectively control the size of Ni nanoparticles through the confinement effect of mesoporous during the reaction. Xu et al. (2013) synthesized a series of ordered mesoporous MgO-Al_2_O_3_ composite oxides with different Mg contents as a support for Ni-based catalysts for CRM reactions. Figure 8 demonstrated a hexagonally ordered mesopore structure of the OM-xMgyAl materials. As shown in Figure 9, the conversion rates of CH_4_ and CO_2_ of 5% Ni/OM-5Mg95Al with mesoporous structure were higher than that of 5% Ni/NP-5Mg95Al with a non-mesoporous structure. Therefore, the mesoporous structure played an important role in improving the catalytic activity of the catalyst. Xu et al. (2016a) compared the catalytic performances of OMA-5Co5Ni, OMA-10Co, OMA-10Ni, and 5Co5Ni/Al_2_O_3_ catalysts toward CRM reaction. As shown in Figure 10, the OMA-5Co5Ni, OMA-10Co, and OMA-10Ni mesoporous catalysts did not suffer significant deactivation after 100-h long-term stability tests due to the confinement effect of the mesoporous framework, which effectively prevented the severe thermal sintering of the metallic Ni and/or Co active centers. In contrast, the traditional 5Co5Ni/Al_2_O_3_-supported catalyst was obviously deactivated because of the seriously thermal sintering of the metallic active sites.

Mesoporous silica materials, such as SBA-15 (Bukhari et al., 2017) and MCM-41 (Tian et al., 2019), are also considered as promising candidates of the catalytic supports owing to their big pore volumes, high surface areas, and excellent thermal stabilities (Chong et al., 2018). Compared with the traditional silica materials, SBA-15 usually possesses an ordered two-dimensional hexagonal mesoporous network with enhanced thermal stability, which can be successfully maintained even after the severe high-temperature reaction conditions (Abdullah et al., 2019; Okutan et al., 2020). Besides, the metallic Ni active sites can be homogenously distributed among the SBA-15 pore channels, which greatly improves the dispersion of active sites over the catalyst surface and mesoporous channels. It is believed that the metal–support interface with strong interaction between the active site and the support can effectively reduce the mobility and thermal sintering of the active site, thereby reducing surface carbon deposition and slowing down the deactivation process of the catalyst (Bukhari et al., 2017). Razak et al. (2019) reported that the Ni/SBA-15 catalyst was synthesized with the oil palm ash as the raw material. The obtained material with the hexagonal ordered mesostructured, small NiO size (16 nm), and strong metal–support interaction performed high conversion rates of CH_4_ and CO_2_. Zhang et al. (2017) prepared ordered mesoporous silica catalysts (Ni-SBA-15, Ni-KIT-6, and Ni-MCM-41) using the solid-phase grinding method. Compared with other catalysts, Ni-SBA-15 showed the best stability in CRM reaction. The high dispersion of Ni species and the strong metal–support interaction between Ni and mesoporous supports reduced the coke deposition and metal sintering of Ni-SNA-15 in the CRM reaction. Yang et al. (2016) prepared a series of Ni/SBA-15-P123 catalysts for the CRM reaction using the P123-assisted impregnation method. The results showed that the SBA-15 support exhibited an excellent confinement effect during the CRM process, which indirectly proved the enrichment of Ni in the mesoporous channels of SBA-15.

In addition, the MCM-41 mesoporous silica also has distinguished structural properties, such as low surface acidity, big pore volume, large specific surface area, high-temperature structural stability, uniform pore-size distribution, and excellent absorption property, which is considered as a suitable catalytic support of the Ni-based catalyst for CRM (Wang X. et al., 2018; Al-Fatesh et al., 2019a). Tian et al. (2019) encapsulated the uniform metallic Ni nanoparticles into MCM-41 straight mesoporous channels driven by the capillary force induced by ethanol. In this way, a limited Ni/MCM-41 catalyst was prepared for CRM reaction. They found that Ni NPs were confined into mesoporous channels with strong metal–support interactions. This confined structure had a significant effect on the inhibition of metal NP agglomeration and coke deposition during the CRM process. Al-Fatesh et al. (2019a) prepared a series of Sc-promoted Ni/MCM-41 catalysts with different loading amounts. The MCM-41 material successfully maintained the mesoporous structure of hexagonal array characteristics after the impregnation of Ni and Sc.

In summary, different supports possess different structures, properties, metal-support interaction, and stability and reducibility of these Ni-based catalysts. Hence, the selection of suitable supports is considered as an important part in the study of Ni-based catalysts for CRM reactions. Compared with conventional supported catalysts, unique structures, such as core–shell structures, can provide relatively superior activity and stability for Ni-based catalysts. Therefore, it is necessary to consider whether they are suitable for large-scale industrial application in the study of Ni-based catalysts with different supports in CRM reaction.

### Ni-Based Catalyst Modified With Different Additives

In order to improve the catalytic performance, various catalytic additives, such as alkaline earth metal oxides, and rare-earth metal oxides, have been extensively investigated toward CRM reaction (Swirk et al., 2020). Meanwhile, doping promoters can also improve the stability of the catalyst by enhancing the surface basicity, redox property, and pore structure of the catalyst by intensifying the coke eliminating process (Singha et al., 2016).

#### Alkaline Earth Metal Oxides

The incorporation of earth metal oxides, such as MgO (Xu et al., 2013) and CaO (Sun et al., 2020), could intensify the surface alkalinity and further intensify the ability of CO_2_ chemisorption and activation on the catalyst surface (Xu et al., 2016a). As a result, the high concentration of CO_2_ on the catalyst surface will make the balance of the Boudouard reaction shift to the left and consequently can reduce the generation of coke deposition (Sun et al., 2019a). Furthermore, the addition of alkaline earth promoters can also affect the degree of reduction and structural property of the catalyst (Ramezani et al., 2018).

**Figure 11:**
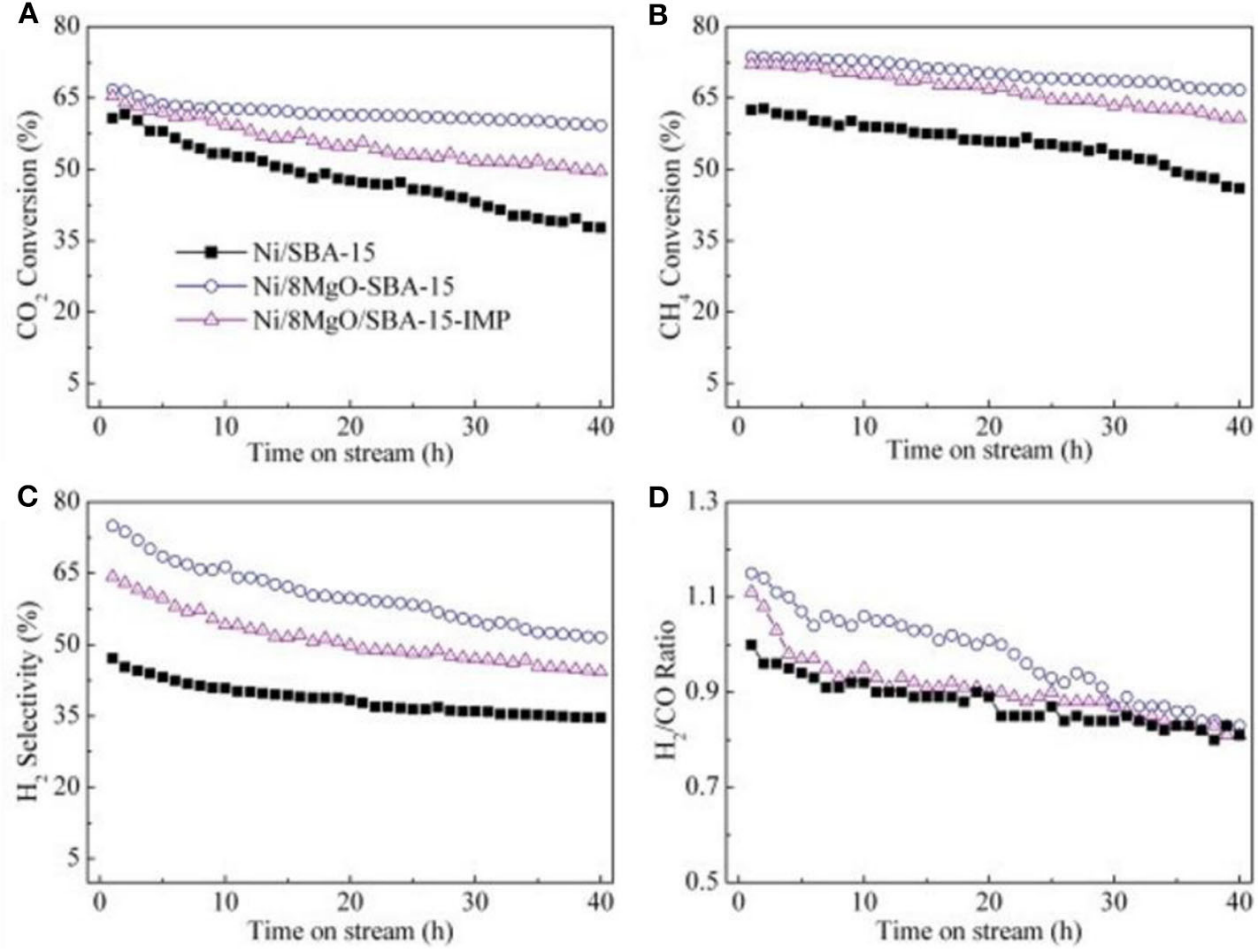
The results of stability test over the samples: **(A)** CO_2_ conversion, **(B)** CH_4_ conversion, **(C)** H_2_ selectivity, and **(D)** H_2_/CO ratio [conditions: GHSV = 36,000 mL/(h·g_cat_), CO_2_/CH_4_ = 1:1, T = 700°C, P = 0.1 MPa]. Reproduced from Wang et al. (2013) with permission from *International Journal of Hydrogen Energy*.

The MgO promoter usually acts as an electron donor, and the electrons in MgO can be transferred to the metallic Ni active sites. In increasing electrons, the density around the metallic Ni could promote the activation and dissociation processes of CO_2_ (Jing et al., in press). It was reported that the addition of MgO can greatly enhance the catalytic activity and coke resistance of the catalyst by improving the dispersion and area of the metallic Ni active site. Besides, it was found that MgO can also effectively improve the activity and stability of the Ni-based catalyst by enhancing the metal–support interaction (Ma et al., 2019). Wang et al. (2013) synthesized the MgO-coated SBA-15 by the one-pot method. The obtained MgO-coated SBA-15 support displayed significantly larger medium basic sites and higher Ni dispersion than the MgO-impregnated SBA-15 counterpart, promising enhanced coke-resistant property and better catalytic stability. As displayed in Figure 11, there was almost no change for the conversions of CO_2_ and CH_4_ over Ni/8MgO-SBA-15 after a 40-h stability test, displaying much excellent stability. Bach et al. (2020) prepared the Ni/Al_2_O_3_ catalyst doped with the MgO promoter (3, 5, and 10 wt.%), performing an effect toward CRM. It was found that MgO could greatly improve the dispersion and metal area of the metallic Ni active site. The Ni/5MgO-Al_2_O_3_ catalyst performs the best anti-coke property and highest H_2_/CO ratio among these catalysts. Singha et al. (2016) compared the catalytic performances of CeO_2_- and MgO-promoted Ni-ZnO_2_ catalysts for CRM. The Ni-ZnO_2_ catalyst promoted by MgO showed better low-temperature activity and coke resistivity than the Ni-ZnO_2_ catalyst promoted by CeO_2_. The high alkalinity of MgO and the excellent redox performance of CeO_2_ increased the dispersion of Ni and formed a strong metal–support interaction, thereby effectively reducing coking on the catalyst surface. Xu et al. (2013) prepared a series of Ni-based catalysts with ordered mesoporous MgO–Al_2_O_3_ composite oxides with different Mg contents as the support for CRM reaction. They found that the MgO basic site was beneficial to enhancing the chemisorption and activation of CO_2_ and improving the catalytic activity of the catalyst.

**Figure 12:**
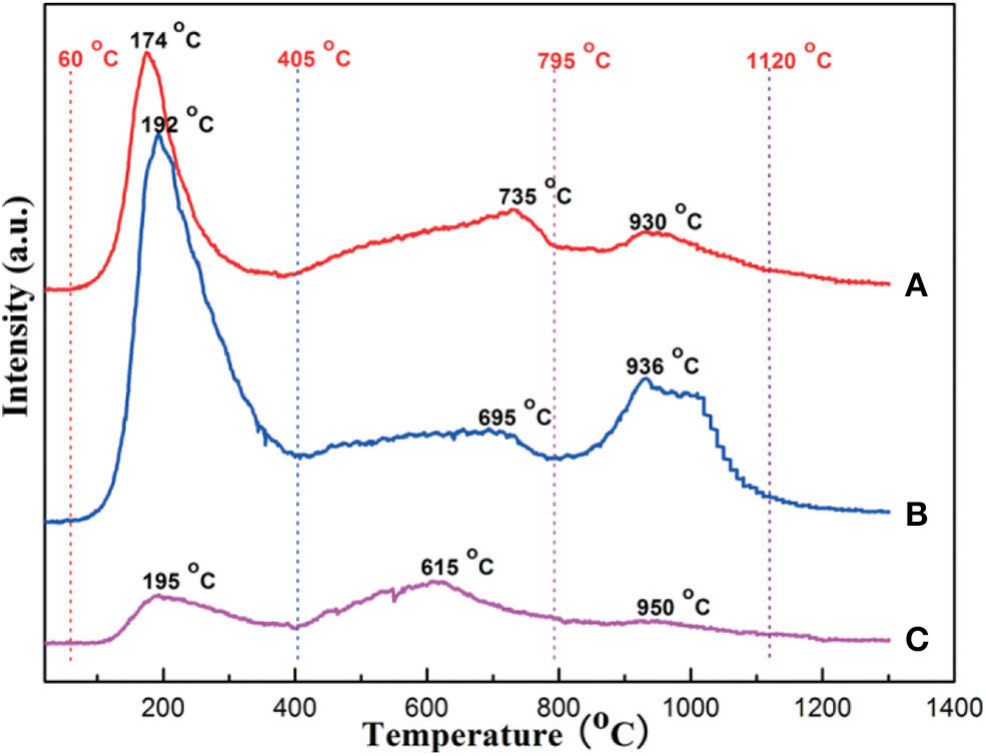
CO_2_-TPD profiles of the catalytic carriers: **(A)** OMA, **(B)** OMA-3Ca, and **(C)** NPA-3Ca. Reproduced from Xu et al. (2014b) with permission from Catalysis Science and Technology.

**Figure 13:**
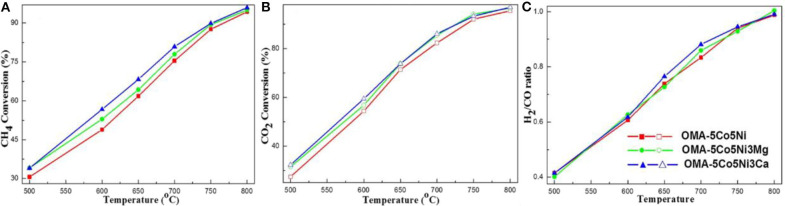
The curves of the **(A)** CH_4_ conversion, **(B)** CO_2_ conversion, and **(C)** H_2_/CO ratio vs. temperature over the catalysts under specific conditions (CH_4_/CO_2_ = 1, GHSV = 15,000 mL g^−1^ h^−1^, 1 atm). Reproduced from Xu et al. (2017) with permission from *Journal of CO*_2_ *Utilization*.

As for CaO, its doping can also improve the chemisorption of CO_2_ and thereby promote the catalytic stability by inhibiting the surface carbon deposition (Xu et al., 2012b). Xu et al. (2014b) synthesized the Ni-based catalyst supported on the ordered mesoporous CaO-Al_2_O_3_ and investigated its catalytic property toward CRM reaction. It could be observed in Figure 12 that the intensity of the CO_2_ desorption peak of OMA-3Ca was stronger than those of NPA-3Ca and OMA because of the addition of Ca, which enhanced the basicity of the OMA-3Ca framework and the number of Lewis basic sites. Xu et al. (2017) fabricated the MgO and CaO promoted ordered mesoporous Co–Ni–MO(=Mg, Ca)–Al_2_O_3_ catalysts. As shown in Figure 13, OMA-5Co5Ni3Mg and OMA-5Co5Ni3Ca displayed much higher CH_4_ and CO_2_ conversions than the reference OMA-5Co5Ni in the range of 500–800°C. This indicated that MgO and CaO basic modifiers could achieve the activation of CO_2_ and CH_4_ at low temperatures. Xu et al. (2012b) synthesized a series of three-compound NiO-CaO-Al_2_O_3_ catalysts with ordered mesoporous structures with different Ca contents. They found that the modification of CaO in the mesoporous framework was also conducive to improving catalytic performance and inhibiting coke deposition by enhancing CO_2_ chemisorption activation. Sun et al. (2020) prepared a series of Ni-Ca-HMS catalysts and investigated them as the catalysts in CRM reaction. Compared with the pristine Ni catalyst, Ca-promoted catalysts exhibited a better coke-resistant property because of their sufficient basic sites and strong metal–support interactions. In addition, the modification of CaO was beneficial to reducing the degree of carbon graphitization, thereby preventing the deposition of coke.

#### Rare-Earth Metal Oxides

The rare-earth metal oxides have been widely considered as the effective additives for the Ni-based catalysts toward CRM reaction (Liang et al., 2020; Muhammad et al., 2020). Rare-earth metal oxides can promote the adsorption of CO_2_ by their basic properties, provide large specific surface areas, and increase catalytic efficiency by increasing surface oxygen vacancies and accelerating charge separation (Tahir et al., 2018).

CeO_2_ is widely used as the promoter for Ni-based catalysts for CRM reaction because of its strong bonding interaction with metallic active site, excellent reduction–oxidation potential (Ce^4+^/Ce^3+^), high oxygen capacity, and abundant activated oxygen species (Tu et al., 2019). Besides, the doping of CeO_2_ could also increase the surface alkalinity of the catalyst, which is conducive to the chemisorption and activation capacity of the reactants, especially CO_2_ (Wang et al., 2020). Akbari et al. (2018) investigated the effect of the Ce loading amount on the catalytic performances of Ni-MgO-Al_2_O_3_ (12.5 wt.% Ni) catalysts by co-precipitation and impregnation methods toward CRM reaction. The CeO_2_-Ni-MgO-Al_2_O_3_ catalyst exhibited much higher activity, better stability, and more excellent anti-coking performance than the Ni-MgO-Al_2_O_3_ catalyst because of the improvement of Ni dispersion and the metal–support interaction by CeO_2_ promotion. Wang et al. (2020) prepared Ni-based activated carbon (NiCexC) catalysts promoted by Ce with different molar contents and investigated the catalytic performance of CRM in non-thermal plasma (NTP) fixed-bed reactors. Among them, the NiCe1C catalyst performed the best catalytic activities and highest product selectivity because the addition of CeO_2_ could form the strong metal–support interaction between Ni and CeO_2_. As a result, the thermal growth of the Ni particles could be effectively inhibited and the activation energy of CH_4_ was reduced due to the size effect. Li and Sibudjing (2018) compared the catalytic performance of multi-Ni core@NiPhy@CeO_2_ shell hollow spheres and Ni@NiPhy without coating of the CeO_2_ shell in CRM reaction. The results showed that Ni@NiPhy@CeO_2_ performed high sintering resistance to both Ni and CeO_2_, promising a high concentration of oxygen vacancies. Thus, it had better catalytic performance and coke resistance to CRM reactions. Li et al. (2011) prepared a CeO_2_-promoted Ni/Al_2_O_3_-ZrO_2_ catalyst by direct sol–gel method. They found that the Ni/Al_2_O_3_-ZrO_2_-CeO_2_ catalyst showed better activity and stability than the Ni/Al_2_O_3_ catalyst because the addition of CeO_2_ effectively improved the dispersion and stability of the Ni particles of the prepared catalyst and enhanced the adsorption of CO_2_ on the catalyst surface.

**Figure 14:**
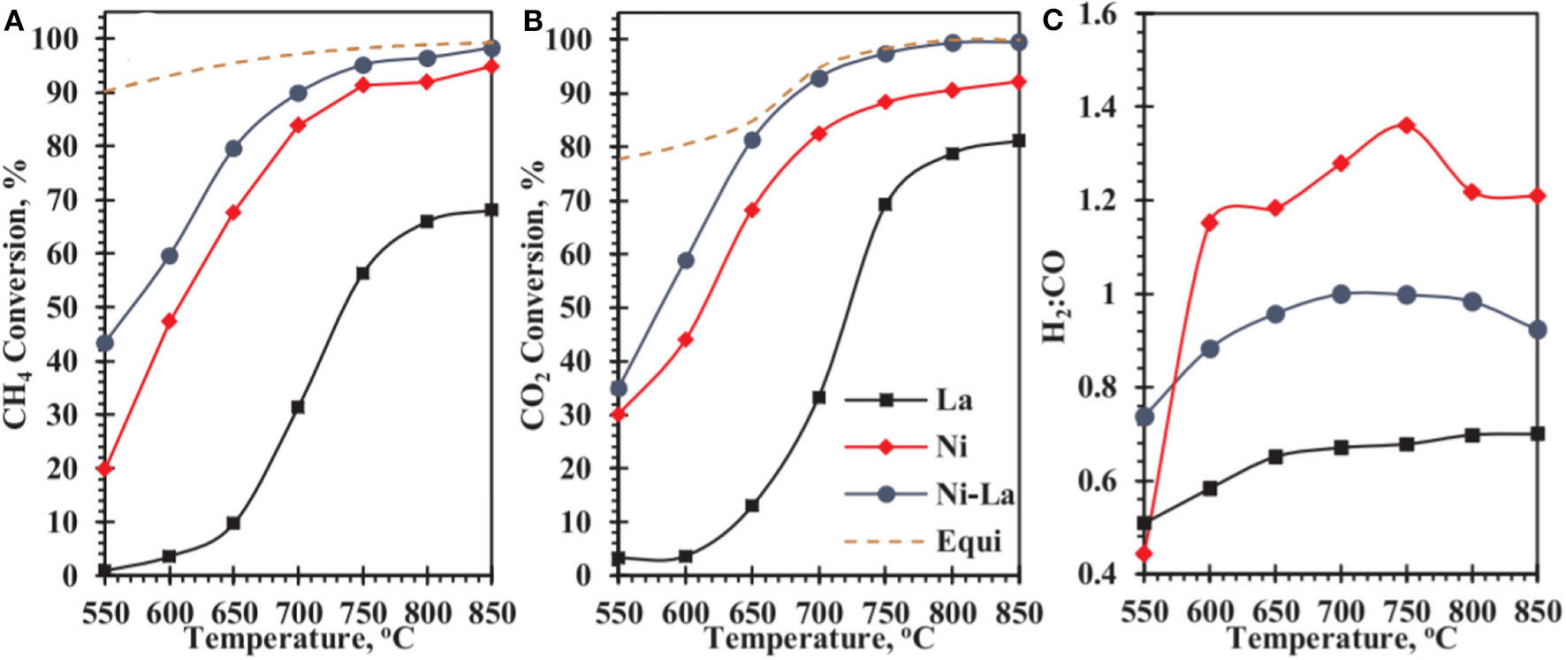
Catalytic conversions of **(A)** CH_4_, **(B)** CO_2_, and **(C)** product H_2_:CO ratio of catalysts as a function of reaction temperature. Reproduced from Abdulrasheed et al. (2020) with permission from *Journal of CO*_2_ *Utilization*.

Recently, the studies revealed that La_2_O_3_ also possessed high oxygen storage capacity and could enhance the metal–support interaction of the catalyst during the CRM process (Wang F. et al., 2016b; Tran et al., 2020). Besides, La_2_O_3_ is also well-known as a strong alkaline oxide, which can intensify the catalyst surface alkalinity. As a result, CO_2_ can be chemisorbed on surface La_2_O_3_ to generate oxidized carbonate (La_2_O_2_CO_3_), which contributed to the elimination of the carbon deposition on the surface of the support (Khoja et al., 2020). Mo et al. (2019) prepared La_2_O_3_-NiO-Al_2_O_3_ catalysts promoted by La_2_O_3_ with different loading amounts to improve the catalytic performance toward CRM reaction. It was found that the doping of La_2_O_3_ could effectively promote the dispersion of metallic Ni and the carbon deposition over the catalyst surface was also further inhibited, accounting for the improved catalytic activity and stability. Abdulrasheed et al. (2020) prepared a Ni–La bimetallic catalyst supported on silica fiber KCC-1 and tested its catalytic activity in CRM. As shown in Figure 14, Ni-La@KCC-1 had higher CH_4_ and CO_2_ conversion rates than single metal Ni or La reference catalysts. The reducibility of NiO crystallites and the dispersion in the fibrous KCC-1 framework was enhanced because of the addition of La_2_O_3_. Thus, more exposed metallic Ni° active sites appeared over the catalyst surface.

In addition to this, many other rare-earth elements are also used as promoters of the Ni-based catalysts for CRM reactions. Al-Fatesh et al. (2019b) synthesized a series of Gd-promoted Ni-based catalysts supported on MCM-41 and tested their catalytic activity for DRM reactions. It was found that Gd not only promoted catalytic activity but also improved the stability of the catalyst. Zhang M. et al. (2020) changed the surface adsorbed oxygen species (SAOS) of the Ni/ZrO_2_ catalyst by doping rare-earth metals such as Ce, La, Sm, and Y. It was proved that the doping of rare-earth metals was beneficial to increasing the amount of SAOS, the Ni dispersion, and the metal–support interaction. The amount of SAOS strongly affected CO_2_ activation and CH_4_ dissociation. Xu et al. (2014a) fabricated a series of functionalized mesoporous Ni–Ln (Ln = Ce, La, Sm, Pr)–Al–O composite oxides. They found that the modification of the rare-earth elements (Ce, La, Sm, Pr) in the mesoporous frameworks could greatly improve the catalytic activity of the catalyst and inhibit coke deposition. They also confirmed that the rare-earth modifiers could affect the distribution of carbon species deposited on the catalyst.

#### Bimetallic Catalysts

The Ni-based bimetallic catalysts have been widely investigated as the catalysts for CRM reaction because of the synergistic effect between Ni and the second metal (Xu et al., 2016a). They are usually prepared by introducing noble or non-noble metals as the second metal. The bimetallic catalyst strategy can not only effectively overcome the drawback of the rapid deactivation caused by carbon deposition over the Ni-based catalyst but also reduce the cost of the catalyst (Niu et al., 2020). The electronic effect produced by metal–metal interaction reduces the sensitivity of the material to carbon deposition (Stroud et al., 2018). Bimetallic catalysts can also increase the dispersion of metallic active sites and reduce the size of metal particles, promising excellent catalytic activity and stability (Erdogan et al., 2018). The Ni-based catalyst is easy to form surface coke during the process of CRM reaction, which causes the decrease of catalyst activity. Therefore, the employment of bimetal or even tri-metal catalysts has been considered as one of the effective methods to improve the coke-resistant capacity of Ni-based catalysts for CRM reaction (Jawad et al., 2019).

It is reported that the addition of the noble metals into Ni-based catalysts can obviously improve the catalytic activity and stability of the catalyst owing to the synergistic effect of active bimetallic catalysts (Niu et al., 2020; Araiza et al., in press). For instance, the strong Ni–Ru interaction of the Ni–Ru bimetallic catalyst could enhance the resistance to carbon deposition because the presence of the Ru was conducive to the gasification of coke and prevented the dissociation of CO (Álvarez et al., 2018). Zhou et al. (2018) confirmed that Ru could accelerate CO_2_ oxidation of surface carbon, enhance the surface oxygen coverage, and slow down the dissociation of CH_4_ during the process of the Ni-MgO-catalyzed CRM, which determined the rate of carbon deposition. For Ni–In bimetal catalysts, Németh et al. (2019) found that the presence of In could change the surface structure of the adsorption site and thus inhibit the complete decomposition of CH_4_ because of the electronic effect on Ni, resulting in the strong chemisorption of H_2_. Therefore, the Ni–In/SiO_2_ catalyst performed much better anti-coking performance than the Ni/SiO_2_ reference catalyst. Pan et al. (2020) synthesized mesoporous silica-supported the bimetallic Ni–Pd catalyst for CRM reaction. The results revealed that the presence of Pd was beneficial to increasing the ratio of the surface-active Ni° of the catalyst. In addition, the doping of Pd and oleic acid was conducive to the formation of small Ni nanoparticles with good dispersion and further inhibiting the thermal sintering.

**Figure 15:**
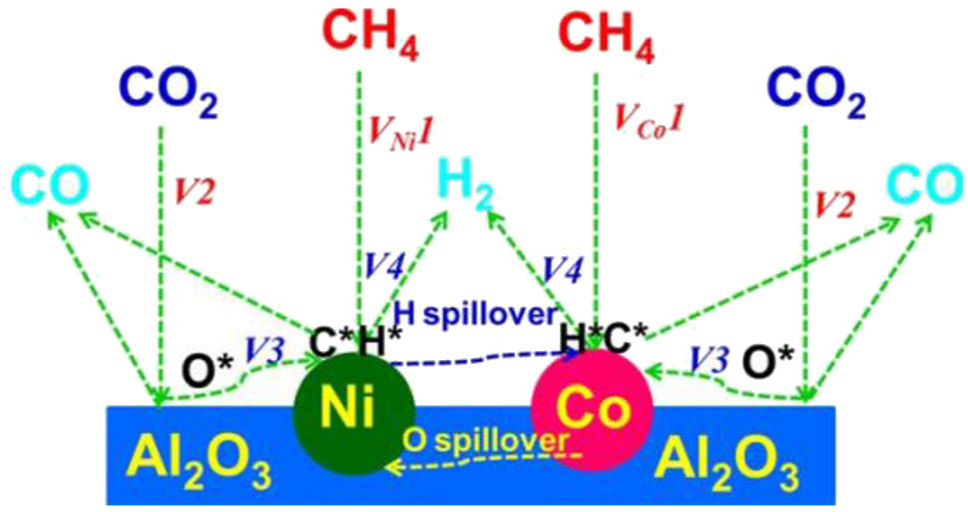
The synergistic effect mechanism in the Co–Ni bimetallic catalyst. Reproduced from Xu et al. (2016a) with permission from ChemCatChem.

In recent years, the Ni–Co bimetallic catalysts have attracted more and more attention because of their outstanding catalytic performances. In the Ni–Co bimetal catalyst, the addition of Co can tune the size of Ni and improve the gasification of coke by enhancing CO_2_ chemisorption, which could reduce the coke yield and further increase the activity and stability of the catalyst (Movasati et al., 2019; Liu et al., in press). Previous reports also found that the Ni/Co atomic ratio is a key factor affecting the catalytic performance and only the appropriate Co/Ni ratio has a synergistic Ni–Co interaction, which improved the catalytic activity and stability of the catalyst (Xu et al., 2016a; Pang et al., 2018). Turap et al. (2020) investigated the effect of Co content on the performance of bimetallic Ni-Co/CeO_2_ catalysts. Their results showed that the catalytic activity over the 0.8Co–Ni/CeO_2_ catalyst was much higher than that over the Ni/CeO_2_ reference catalyst toward CRM reaction. This was attributed to the strong adsorption of oxygen over the Ni–Co alloy and the redox property of CeO_2_ support, which could directly break the carbon–oxygen bond and inhibit the occurrence of the RWGS reaction. Erdogan et al. (2018) compared the activities of Ni-, Co-, and Ni–Co-based catalysts supported on the SBA-15 toward CRM reaction. The 4Ni-1Co@SBA-15 catalyst performed the highest CO and H_2_ selectivity among these catalysts, indicating that the incorporation of Co had a significant effect on the catalytic activity of the Ni catalyst. The catalytic activity of the 4Ni-1Co@SBA-15 bimetallic catalyst was better than those of the Ni and Co single metal catalysts because of the formation of Ni–Co alloy. Xu et al. (2016a) prepared a series of Co–Ni bimetallic catalyst-doped ordered mesoporous Al_2_O_3_ composites oxides. Due to the synergistic effect of Co–Ni, ordered mesoporous Co–Ni bimetallic catalysts exhibited higher catalytic activity and better coking resistance than the corresponding Ni and Co monometallic counterpart catalysts. The specific synergistic effect mechanism in the Co–Ni bimetallic catalyst was illustrated in Figure 15.

It was reported that the Ni–Sn interaction is essential to the achievement of the good catalytic activity (Stroud et al., 2018). This is because Sn atom has an electronic structure similar to C atom. This will facilitate the interaction between the p orbital of Sn and the 3d electron of Ni and thereby slow down the formation of coke (Guharoy et al., 2018). In addition to this, Sn can also improve the dispersion of the metallic Ni and promote the oxidation of key reaction intermediates on the surface of the catalyst. Thereby, the formation of the final product will be facilitated (Bobadilla et al., 2016; Guharoy et al., 2018). Stroud et al. (2018) found that the addition of Sn could greatly promote the catalytic activities and stabilities over the investigated Ni-Sn/Al_2_O_3_ and Ni-Sn/CeO_2_-Al_2_O_3_ catalysts toward CRM reaction. As a result, the process of the coke formation was slowed down because the Sn atom could occupy the neighboring C-shaped nucleus site on the Ni atom. However, an excessive amount of Sn would exert the adverse effect on the catalytic performance, owing to the coverage of the Ni active site, which limited the access of the gaseous reactants and thereby reduce the conversion of reactants.

In general, adding alkali metal and alkaline earth metal oxides can increase the surface basicity of the catalyst and thus increase the amount of CO_2_ adsorbed on the catalyst surface and improve the carbon removal effect of CO_2_. The addition of La_2_O_3_ and CeO_2_ mainly enhances the ability of oxygen adsorption and dissociation, inhibit the generation of carbon deposits, and improve the antioxidant capacity of the active component Ni. Therefore, choosing appropriate additives plays a vital role in the catalytic performance of the Ni-based catalyst. Bimetallic catalysts exhibit unique properties and excellent catalytic performance, which are different from single metal catalysts due to their adjustable physical and chemical properties, electronic, and geometric effects between bimetals. Although the bimetallic catalysts with noble metals have good catalytic activity and selectivity, the non-metallic doped bimetallic catalysts have a wider application prospect because of the advantage of cost.

## Preparation Method

The preparation method plays a crucial role in affecting the performances of the catalysts toward the CRM reaction. This is because it can affect the composition of the catalyst, particle size, dispersion of the active sites, and surface interaction between catalyst components (Hassani Rad et al., 2016). These properties could greatly affect the catalytic performances of CRM at high temperature by enhancing their anti-sintering and anti-coking properties (Hambali et al., 2020; Shah et al., 2020). Generally, impregnation method (Zou et al., 2017), sol–gel method (Aghamohammadi et al., 2017), and co-precipitation method (Zhang G. et al., 2019) are common methods for synthesizing the Ni-based catalysts. Specifically, the impregnation method usually puts the support into the solution with dissolved active components, and the solution enters the pore channels of the support by capillary pressure (Romero-Sáez et al., 2018). The impregnation method is easy to operate and control the dispersion of the metallic active sites on the support. However, it can only achieve the small loading amount because of the limitation of the saturation solubility of the metal precursor and the metal–support interaction between the Ni nanoparticles and the support is weak (Arbag, 2018). It was reported that the impregnation sequence of metal precursors could greatly affect the metal-support interaction over the polymetallic catalysts prepared by impregnation, which further had a certain effect on the performance of the catalyst (Zou et al., 2017). Romero-Sáez et al. (2018) prepared the Ni-ZrO_2_ catalysts supported on CNT by the sequential impregnation and co-impregnation methods. It was found that the NiO nanoparticles were wrapped by ZrO_2_ in the core–shell structure over the catalyst prepared by co-impregnation method and the NiO nanoparticles were on or near the surface of ZrO_2_ over the catalyst prepared by the sequential impregnation method. Arbag (2018) reported that the impregnation sequence of Mg had a great influence on the activity of Ni-based catalysts supported on mesoporous alumina. The catalyst subsequently impregnated with Mg and Ni could achieve the highest coking rate. As a comparison, the catalyst synthesized by co-impregnation of Mg and Ni almost behaved no coking phenomenon because of the strong interaction between Ni and Mg by forming NiO–MgO solid solution at high calcination temperature.

**Figure 16:**
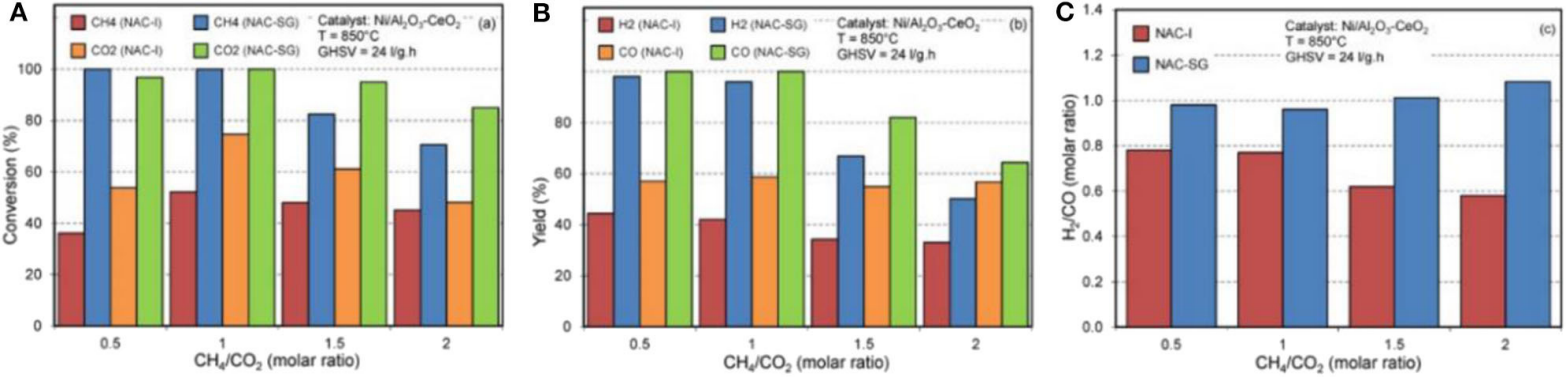
Effect of synthesis method on **(A)** feed conversion, **(B)** products yield, and **(C)** H_2_/CO molar ratio in a product at different feed ratios over Ni/Al_2_O_3_-CeO_2_ nanocatalyst. Reproduced from Aghamohammadi et al. (2017) with permission from Molecular Catalysis.

In case of the sol–gel method, it can quickly achieve molecular homogeneity of the active sites because they can be individually mixed on the molecular level during the formation of gel (Tao et al., 2013). During the sol–gel process, it is easy to achieve the uniform doping of the active ingredients in molecular level at low synthesis temperature (Aghamohammadi et al., 2017). However, the sol–gel process usually takes a long time and a large amount of gas and organic compounds will be released during the drying process (Lv et al., 2020). The catalysts prepared by the sol–gel method possess the advantages of narrow particle size distribution, high resistant property to thermal agglomeration, and low deactivation rate (Hassani Rad et al., 2016). Aghamohammadi et al. (2017) prepared a Ni/Al_2_O_3_-CeO_2_ nanocatalyst by the sol–gel method. As shown in Figure 16, the NAC-SG catalyst displayed much higher feed conversion, product yield, and H_2_/CO ratio than NAC-I catalyst. Marinho et al. (2020) found the positive effect of the sol–gel method on the stabilization of Ni particle size in the reduction process at high temperature (800°C) Ni@CeO_2_. Besides, the above catalysts prepared by the sol–gel method also generated more oxygen vacancies and stronger metal-support interaction with CeZrO_2_ and Ni than the impregnated Ni/CeO_2_ catalyst. Shin et al. (2018) reported that the Ni/ZrO_2_-Al_2_O_3_ catalyst prepared by Pechini sol–gel method showed higher stability than those synthesized by urea hydrolysis and physical mixing methods due to the strong metal–support interaction between Ni and ZrO_2_-Al_2_O_3_, which further promoted the dissociation of CO_2_.

**Figure 17:**
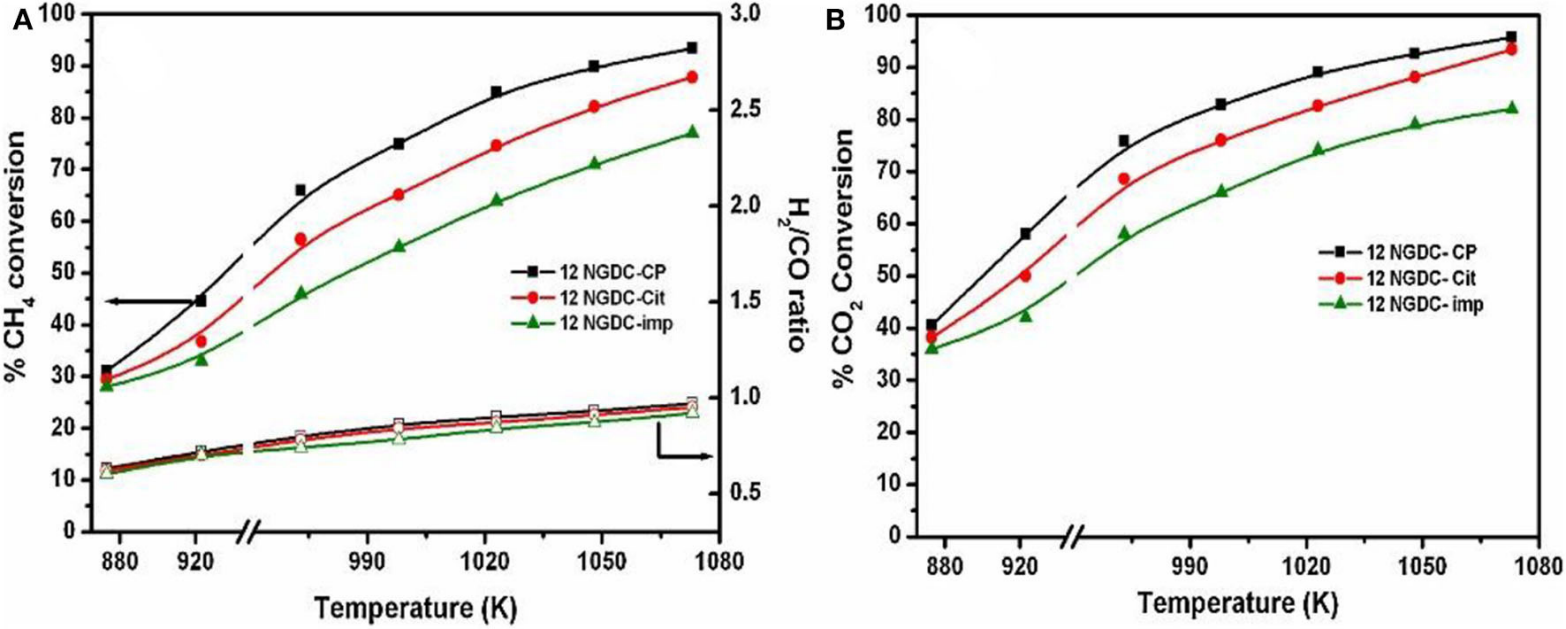
Effect of temperature on various 12NGDC catalysts, **(A)** CH_4_ conversion and H_2_/CO ratio and **(B)** CO_2_ conversion. Reaction conditions: CH_4_:CO_2_:N_2_ = 1:1:1, GHSV = 28,800 h^−1^ (filled symbols-conversion, hollow symbols-H_2_/CO ratios). Reproduced from Gurav et al. (2017) with permission from *Journal of CO*_2_ *Utilization*.

As for the co-precipitation method, it usually has many advantages, such as simple preparation process, low cost, easy preparation control, and short synthesis time. Therefore, it has been one of the most widely investigated preparation methods (Pejjai et al., 2020). However, the agglomeration or uneven composition usually takes place because the addition of the precipitant may make part of the concentration too dense (Marzano et al., 2019). Therefore, how to improve the activity and the resistance to carbon deposition over the Ni-based catalyst by employing unconventional preparation methods is still a challenge. Gurav et al. (2017) prepared the Gd-doped Ni-based catalyst supported on CeO_2_ by the co-precipitation method, citrate gel method, and impregnation method. As could be observed in Figure 17 12NGDC-CP performed the highest conversion rates of CH_4_ and CO_2_ among these three catalysts because of the highest metal dispersion. Therefore, the preparation method can obviously affect the metal dispersion and then influence the catalytic activity.

In recent years, the plasma method has attracted more and more attention because of its unique advantages, such as compact structure, strong material adaptability, and fast response (Snoeckx et al., 2015; Biset-Peir et al., 2020). Among the plasma techniques, the low-temperature plasma is commonly used for the preparation of the Ni-based catalysts toward CRM reaction. The advantage of the low-temperature plasma is that it can activate the gas through electron collisions to stimulate ionization and dissociation reactions rather than heat the entire reactor (Chai and Kwon, 2019). It was reported that the dielectric barrier discharge plasma (DBD) decomposition is an excellent method of preparing Ni-based catalysts with improved activity toward CRM reaction (Hu et al., 2019). Hu et al. (2019) investigated the Ni/ZrO_2_ catalysts prepared by traditional calcination (NiZrO_2_-C) and plasma (Ni/ZrO_2_-P) methods for the CRM reaction. Compared with NiZrO_2_-C, the Ni/ZrO_2_-P catalyst displayed more Ni(111) planes, smaller metallic Ni particles, and more oxygen vacancies. As a result, the Ni/ZrO_2_-P catalyst displayed better catalytic activity than Ni/ZrO_2_-C toward CRM reaction. Fang et al. (2016) investigated the catalytic activity of Ni/Y_2_Zr_2_O_7_ catalysts treated by DBD toward CRM reaction. They found that plasma treatment could enhance the metal–support interaction of Ni/Ni-Y_2_Zr_2_O_7_, generate small NiO nanoparticles, and produce a large metallic Ni active surface area. Thereby, the Ni/Y_2_Zr_2_O_7_ catalyst with the highest activity, the best stability, and the strongest coke resistance can be obtained by plasma treatment in H_2_/Ar mixture gas stream.

Some other catalyst preparing methods, such as homogeneous deposition (Park et al., 2019), combustion synthesis (Gonzalez-Delacruz et al., 2010), reflux digestion (Chen et al., 2013), and atomic layer deposition (ALD) (Zhao et al., 2018), are also investigated to synthesize the Ni-based catalysts for CRM reactions. As for the homogeneous precipitation method, the choice of precipitants has a great impact on the catalytic performance of the catalyst. Zhang G. et al. (2019) used alkaline precipitants to obtain Ni_3_Si_2_O_5_(OH)_4_ nanosheets in the catalyst precursor. Among them, the strong electrolytic capacity of NaOH made the most Ni_3_Si_2_O_5_(OH)_4_ nanoflakes formed in the Ni-MSC-1 catalyst, which results in the highest Ni dispersity and highest catalytic activity. Park et al. (2019) studied the effect of preparation methods on the catalytic performance of Ni-substituted CaZrO_x_ catalysts toward CRM reaction. Compared with Ni/CaZrO_x_ prepared by the impregnation method, CaZrNiO_x_ prepared by the homogeneous deposition method behaved in a larger number of alkaline sites, higher surface area, better Ni dispersion, and stronger metal–support interaction. Therefore, CaZrNiO_x_ demonstrated better catalytic activity and stability during the CRM reaction. Gonzalez-Delacruz et al. (2010) prepared the Ni-CeO_2_ catalyst effectively under various metal loadings through a combustion synthesis method, which was conducive to controlling the final stoichiometry and quantity production of catalysts without complicated equipment, separation process, and expensive precursors. Chen et al. (2013) prepared the Ni-CaO-ZrO_2_ nanocomposite catalyst by the co-precipitation method and reflux-digestion method. They found that reflux digestion conditions had a significant effect on the catalyst structure, crystal phase, and catalytic performance. Higher reflow temperature and longer reflow time were beneficial to the formation of smaller particles. The ALD method is widely known for its ability to form films with high uniformity, consistency, repeatability, and thickness accuracy at the atomic level (Shang et al., 2017). Zhao et al. (2018) developed a novel Al_2_O_3_/Ni/Al_2_O_3_-sandwiched catalyst by the ALD method. They found that the dual interactions of Ni with γ-Al_2_O_3_ support and Al_2_O_3_ film could effectively inhibit Ni aggregation at high temperature and subsequent carbon deposition. The catalyst showed excellent catalytic activity and stability in CRM reaction and displayed good industrial application potential.

In general, each preparation method has its own advantages in the process of preparing the catalyst and the performance of the prepared catalyst. Even for the same active component, the difference in preparation method will affect the reducing particle size dispersion of the active component and the microstructure of the catalyst, which will cause the significant difference in the reaction activity and selectivity of the catalyst, as well as the significant difference in the anti-carbon deposition ability. Therefore, the catalytic performance of the catalyst can be tuned by controlling the difference in the preparation method. Meanwhile, the new preparation method provides more possibilities for improving the catalytic performance of the Ni-based catalyst toward CRM reaction.

## The Mechanism of CRM

**Figure 18:**
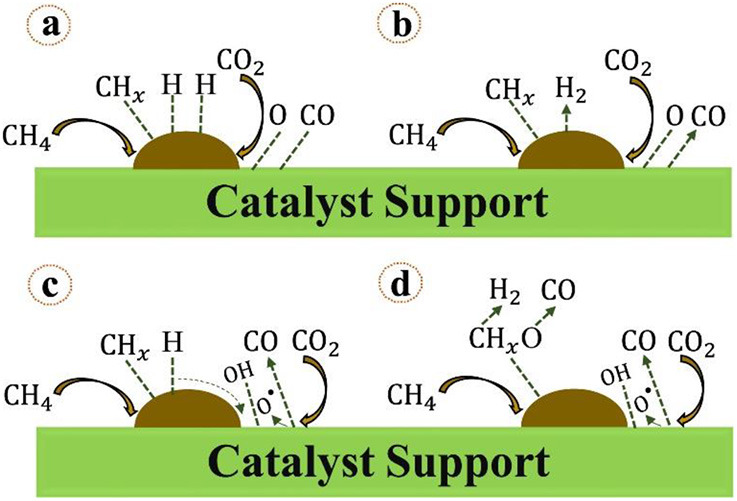
Mechanistic steps for methane reforming reaction involving: **(A)** chemisorption and dissociation of CH_4_ and CO_2_ on metal-support interface, **(B)** fast rate of H_2_ and CO desorption, **(C)** hydrogen and oxygen spillover forming surface hydroxyls, **(D)** oxidation of hydrogen-depleted CHx species by surface hydroxyls and oxygen groups to produce H_2_ and CO. Reproduced from Abdulrasheed et al. (2019) with permission from Renewable and Sustainable Energy Reviews.

With the recent development and wide application of the experimental characterization, the mechanism of DRM reaction has been extensively investigated. Abdulrasheed et al. (2019) illustrated the scheme of the CRM mechanism of CRM. It can be generally summarized into the following steps: dissociative adsorption of CH_4_, dissociative adsorption of CO_2_, hydroxyl group formation, intermediate oxidation, and desorption, as shown in Figure 18.

The CRM reaction process is accompanied by multiple side reactions. Various catalytic systems and process conditions will possess different catalytic mechanisms. So far, the global scholars generally recognize that the process of CH_4_ conversion over the metallic active site mainly contains the following steps: (1) the gradual decomposition and dehydrogenation of CH_4_ to ${\text{CH}}_{\text{x}}^{*}$(0 < x <4) and H^*^, (2) the recombination of H^*^ to generate H_2_, and (3) the desorption of the catalyst surface at high temperature (Zhou et al., 2018). However, the viewpoints of the CO_2_ conversion mechanism are controversy between different researchers. Some researchers believe that CO_2_ is chemisorbed on the metallic active site or catalytic support to form the carbonate. Subsequently, the activated carbonate further reacts with ${\text{CH}}_{\text{x}}^{*}$ to generate CO and H_2_ (Wang et al., 2014; Foppa et al., 2016). Another viewpoint of the CO_2_ activation is that CO_2_ can be directly dissociated into CO and O^*^ on the metallic active site of the catalyst. Then, O^*^ reacts with ${\text{CH}}_{\text{x}}^{*}$ to generate CO and H^*^ (Zhu et al., 2009).

**Figure 19:**
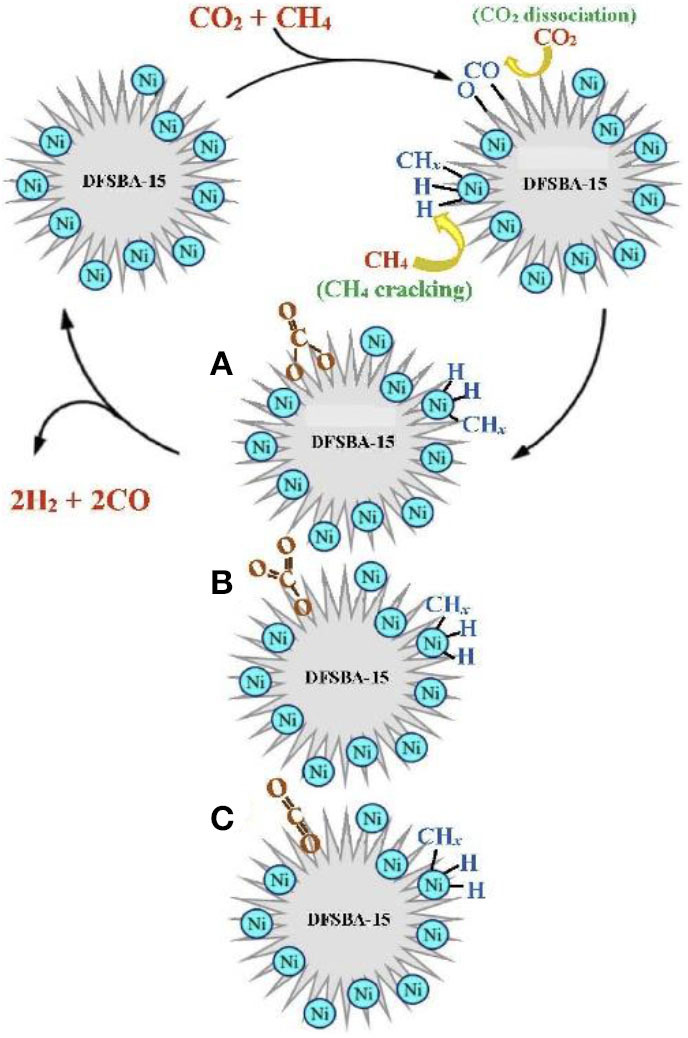
Reaction scheme of Ni/DFSBA-15 over CRM. **(A)** Bidentate carbonate, **(B)** unidentate carbonate, and **(C)** linear carbonyl. Reproduced from Chong et al. (2020) with permission from *International Journal of Hydrogen Energy*.

**Figure 20:**
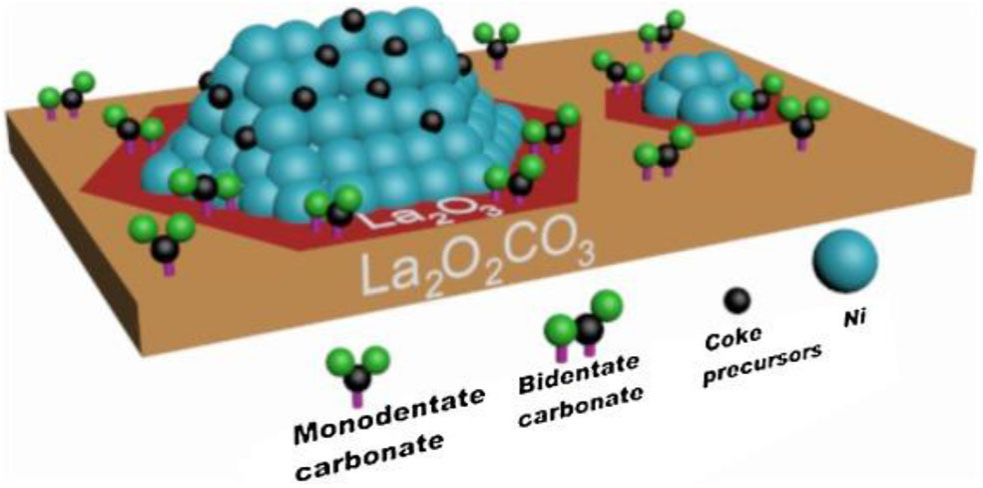
Proposed Ni/La_2_O_3_ catalyst model in the DRM process. Reproduced from Li K. et al. (2019) with permission from *Applied Catalysis B: Environmental*.

**Figure 21:**
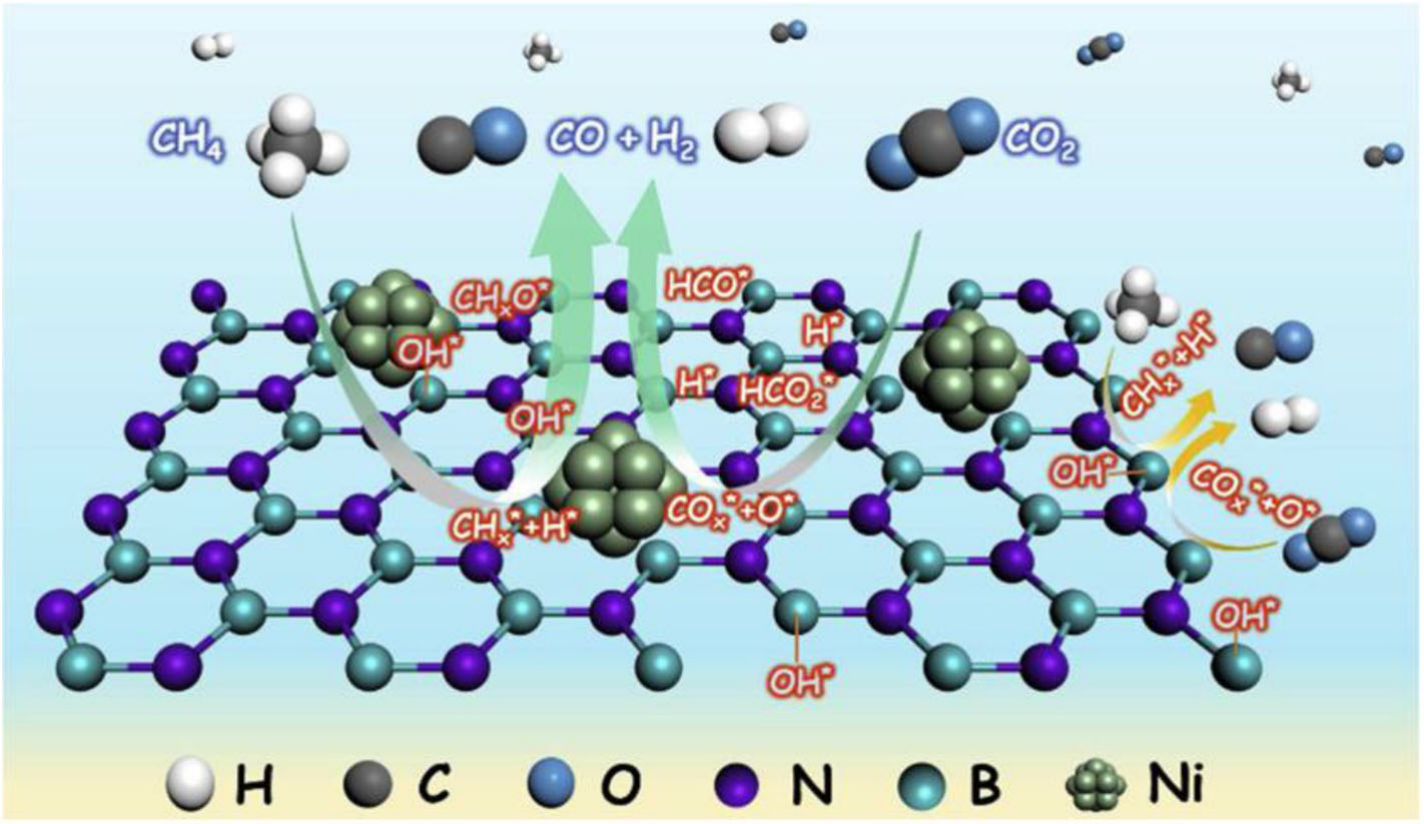
Possible reaction mechanism over Ni/d-BN catalysts. Reproduced from Bu et al. (2020) with permission from *Applied Catalysis B: Environmental*.

**Figure 22:**
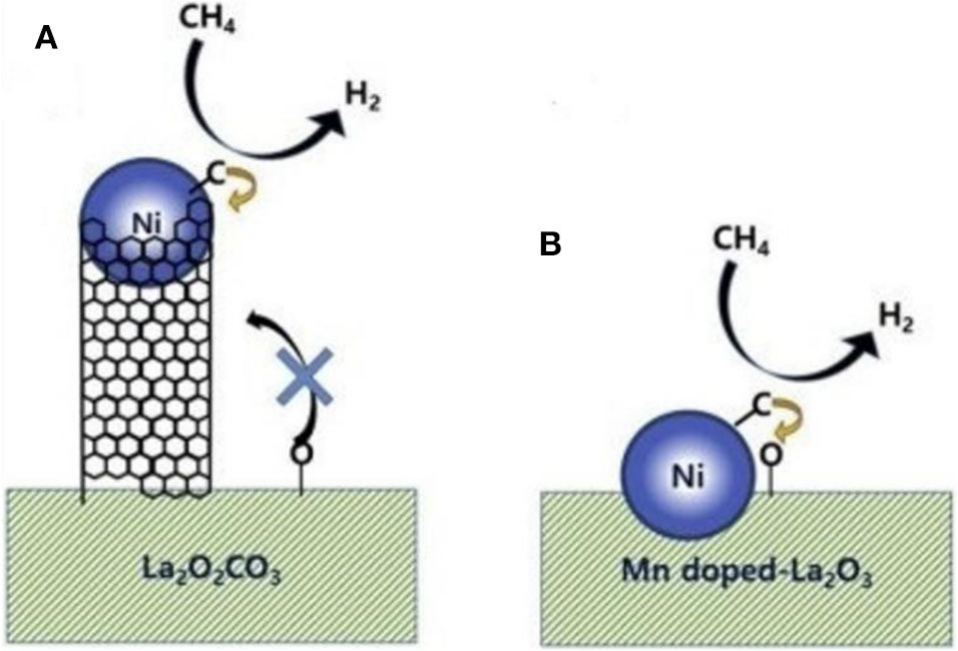
A schematic model of catalyst deactivation by coking. **(A)** Active metal nanoparticles depart from La_2_O_2_CO_3_ support by filamentous carbon growth that blocked oxygen supply to the metal surface. **(B)** The metal nanoparticles maintain close contact with the support to receive steady oxygen supply to produce CO. Reproduced from Kim et al. (2019) with permission from *Applied Catalysis A: General*.

Chong et al. (2020) reported the mechanism of syngas production through CRM of 10Ni/DFSBA-15 as shown in Figure 19. CO_2_ tended to dissociate into CO^*^ and O^*^ or adsorb on the catalyst surface and then interact with the basic oxygen atoms on the surface to form intermediate species, such as unidentate carbonate, bidentate carbonate, and linear carbonyls, during the CRM reaction over the 10Ni/DFSBA-15 catalyst. Subsequently, these reaction intermediates further reacted with CH_4_ molecules to generate H_2_ and CO. Li K. et al. (2019) found that the interface between Ni and La_2_O_3_ promoted the formation of bidentate carbonate and further participated in the coke removal process over the Ni/La_2_O_3_ catalyst. Therefore, the reaction mechanisms and scheme of catalyst models was assumed as shown in Figure 20. The activation and decomposition of CH_4_ occurred on the Ni particle surface to generate activated coke precursors and H_2_. CO_2_ was adsorbed on the interface between Ni and La_2_O_3_ to forms bidentate carbonate. Bidentate carbonate reacted with adjacent activated coke precursors to form CO. During this process, it was observed that the strength of bidentate carbonate gradually decreased while the strength of monodentate carbonate remained stable. This indicates that the bidentate carbonate could react effectively with the coke intermediate. Moreover, it was found that the activation of methane and the formation of CO took place at the same time, demonstrating that CO_2_ participated in the coke-elimination reaction pathway and reacted with carbon species to inhibit coke deposition. They also found that the addition of Ni promoted the formation of bidentate carbonate on the La_2_O_3_ catalyst and the 5Ni/La_2_O_3_-m catalyst performed a strong promotion effect on the formation of bidentate carbonate intermediate. Bu et al. (2020) prepared the Ni/d-BN with defect confinement toward CRM reaction. The mechanism for the DRM reaction for Ni/d-BN was displayed in Figure 21. Firstly, CH_4_ and CO_2_ dissociate into CHx^*^, H^*^, O^*^, and CO^*^ and other active species at the defect sites of d-BN and the interface between Ni and d-BN. Subsequently, the presence of H^*^ can further facilitate the activation of CO_2_ and assist its conversion into –HCO_2_. Then,–HCO_2_ will be decomposed into –HCO and O^*^. Besides, H^*^ and O^*^ can easily combine into –OH at the B terminal of d-BN, and –OH can assist ${\text{CH}}_{\text{x}}^{*}$ conversion into formyl (–HCO) species, and –HCO can rapidly decompose into CO and H^*^. Because of the presence of H^*^ and O^*^ in the reaction, even if –OH is consumed by CHx^*^, H^*^ and O^*^ can rapidly bind to B terminal sites to reform B–OH species. Kim et al. (2019) studied the catalytic performance of the reduced Co- and Mn-modified perovskite LaNiO_3_ catalyst toward CRM reaction. They found that CO_2_ could chemisorb on the La_2_O_3_ support and then generate the oxygen atom. CH_4_ could be dehydrogenated on the surface of metallic Ni (or Co) sites to generate carbon atom and hydrogen atom. Finally, the oxygen atom reacted with the carbon atom to produce CO. Furthermore, it could be observed in Figure 22 that the formation of coke was confirmed. The metallic active nanoparticles were separated from La_2_O_2_CO_3_ by inhibiting the oxygen supply from the filamentous carbon chains to the metal surface. The metal nanoparticles were kept in close contact with the supporting site to obtain a stable oxygen supply and CO generation. When a large amount of coke was formed, the metal particles were removed from the scaffold by filamentous carbon. As a result, there was no oxygen supply from La_2_O_3_. The carbon atoms formed on the metal surface could not be effectively eliminated and the polymerization of the carbon atoms accelerated the coking process. Therefore, the close contact between the metallic active site and the support had an important impact on the high activity and stability of the CRM catalyst because the CO formation reaction might take place at the metal–support interface. In summary, so far there has not been a single reaction mechanism that can summarize all CRM reactions. However, extensive research on CRM reaction mechanism contributes to a deeper understanding of CRM reaction and then design of the catalyst with better catalytic activity.

## Conclusions and Perspective

CO_2_ has been considered as one of the major incentives of many environmental problems, such as global warming and extreme weather. CRM not only effectively utilizes the CO_2_ resource and reduce carbon emissions but also produces the value-added syngas, which can be further employed as the building unit of the synthesis of alcohols, olefins, and other valuable products. For now, the main challenge of the CRM reaction is to develop a highly efficient catalyst with high catalytic activity, sinter proof, coke resistance, and low cost.

Ni-based catalysts have been widely regarded as promising alternatives to noble metal catalysts. However, their industrial application is limited due to the serious sintering of Ni nanoparticles and rapid catalyst deactivation caused by coke deposition. Various strategies of improving the performance of catalysts are carefully and comprehensively summarized in this review. In order to reduce carbon deposition on Ni-based catalysts, researchers have tried to add promoters to change the alkalinity of the support, such as alkaline earth metal oxides and rare-earth metal oxides, which can enhance the stability and decrease carbon formation. It is found that the catalytic performances can be affected by the preparation methods. Besides, the choice of the support is also very crucial. Various materials, such as metal oxides, ordered mesoporous silica gel materials, zeolite materials, and carbon materials, can be used as the supports of the Ni-based catalysts.

In future research, it can be prospected that the research hotspot of Ni-based catalysts in the field of CRM will focus on the Ni-based catalysts supported or confined by novel materials, such as ordered mesoporous materials and hollow zeolites. Specifically, the mesoporous framework in these catalysts facilitates the dispersion of active sites, and the confinement effect of mesoporous channels or hollow cavity can effectively control the size of Ni nanoparticles during CRM reactions. Besides, the combination of CRM with other reactions, such as SRM and POM, can alter the H_2_/CO ratio of syngas by changing the feedstock gas ratio, which will provide different approaches for large-scale industrial applications of CRM.

## Author Contributions

LX conceived of the presented idea. LX encouraged XWu to investigate the project and supervised the whole progress of this work. CL, YC, XWen, CW, BY, and ZM assisted XWu to carry out the literature survey and summary. XWu, LX, and MC wrote the manuscript with support from XH. LX, MC, and XH supervised the whole project. All authors discussed the results and contributed to the final manuscript.

## Conflict of Interest

The authors declare that the research was conducted in the absence of any commercial or financial relationships that could be construed as a potential conflict of interest.
